# Nutritional disorders have adverse effect on clinical outcomes and may increase healthcare costs following hip and knee arthroplasty—A systematic review

**DOI:** 10.1002/jeo2.70657

**Published:** 2026-10-15

**Authors:** Christopher David John Little, Hariharan Subbiah Ponniah, Shvaita Ralhan, Louise Strickland, Antony Palmer, Thomas William Hamilton

**Affiliations:** ^1^ Nuffield Department of Orthopaedics Rheumatology and Musculoskeletal Sciences (NDORMS) Oxford UK; ^2^ Oxford University Hospitals NHS Foundation Trust Oxford UK; ^3^ Imperial College Healthcare NHS Trust London UK

**Keywords:** arthroplasty, frailty, malnutrition, nutritional disorder, outcome, sarcopenia, systematic review

## Abstract

**Purpose:**

To report the effect of nutritional disorder on outcomes following hip and knee arthroplasty using European Society of Clinical Nutrition and Metabolism (ESPEN) diagnostic criteria.

**Methods:**

A systematic review of level 1–4 evidence was conducted as per the PRISMA statement and Cochrane Handbook for Systematic Review (PROSPERO ID: CRD42023360517). In March 2024, AMED, CENTRAL, EMABASE, MEDLINE, Scopus and Web of Science were searched. Articles were included if they defined and reported the effect of nutritional disorder on outcome(s) of interest following hip and/or knee arthroplasty. Exclusion criteria were pathological fracture and <50 cases. The ROBINS‐I was used to assess study bias. Data were presented with effect sizes if reported.

**Results:**

Fifty‐eight studies were included (2,076,289 patients). Malnutrition, as per ESPEN criteria, increased mortality, post‐op pain and length of hospital stay. Sarcopenia, diagnosed via computed tomography, increased surgical site infection rate following primary arthroplasty and mortality following hip fracture arthroplasty. Frailty worsened clinical outcomes following primary, revision and hip fracture arthroplasty. One included study reported healthcare cost; these were higher among frail patients undergoing primary total knee arthroplasty: $19,841 versus $6104, *p* = 0.001.

**Conclusion:**

Nutritional disorder was associated with adverse clinical outcomes following primary, revision and hip fracture arthroplasty. Moderate‐to‐severe bias, heterogenous definitions of nutritional disorder and lack of standardised outcome reporting limited analyses, particularly analyses of healthcare costs which likely increase with nutritional disorder. Prospective study is required to examine potential for cost‐saving and improved patient outcome following treatment of nutritional disorder in hip and knee arthroplasty populations.

**Level of Evidence:**

Level IV.

## Graphical abstract

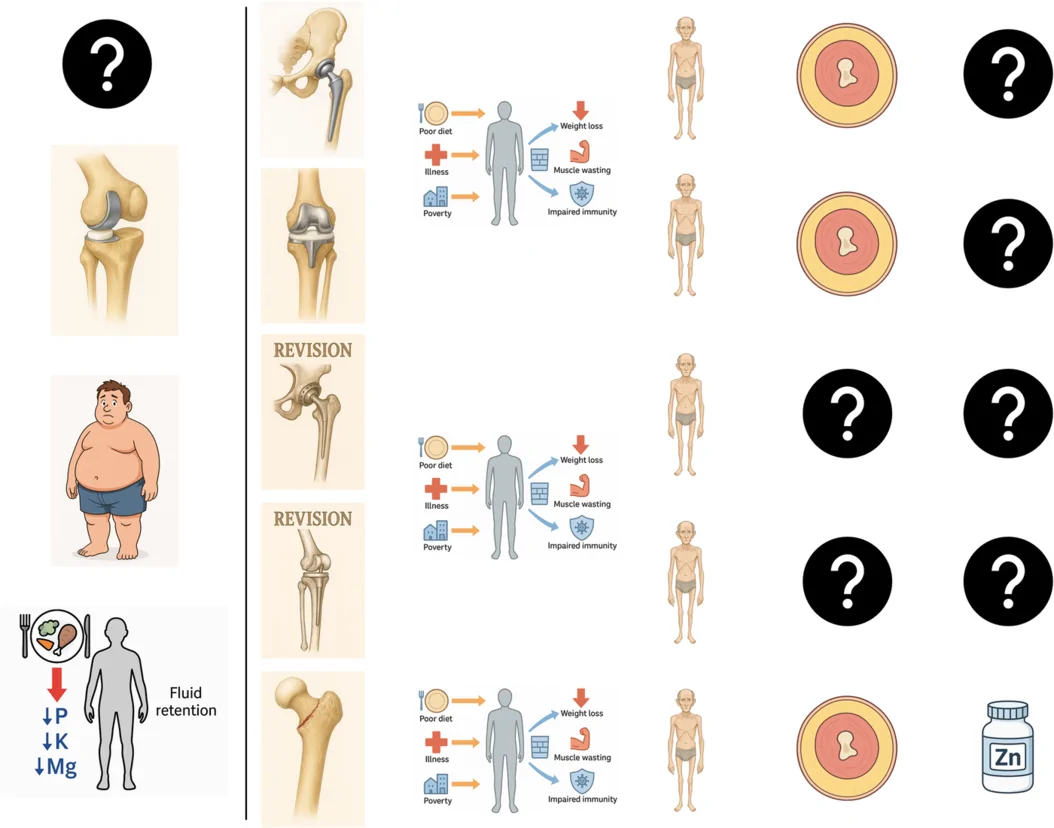

**Abbreviations**
ADL: activity of daily living
ASPEN: American Society for Parenteral and Enteral Nutrition
BMI: body mass index
CGA‐FI: Comprehensive Geriatric Assessment‐Frailty Index
CPRD: Clinical Practice Research Database
CT: computed tomography
eFI: Electronic Frailty Index
EQ. 5D: EuroQoL ‐ 5 Dimension
ESPEN: European Society of Parenteral and Enteral Nutrition
FI: Frailty Index
GFI: Groningen Frailty Indicator
HFRS: Hospital Frailty Risk Score
HOOS: Hip Osteoarthritis Outcomes Score
KOOS: Knee Osteoarthritis Outcomes Score
LOHS: length of hospital stay
MCID: minimal clinically important difference
MFI: Modified Frailty Index
MNA: mini nutritional assessment
MUST: Malnutrition Universal Screening Tool
NHS: National Health Service
NRS: Nutritional Risk Score
NSQIP: National Surgical Quality Improvement Program
PJI: Prosthetic Joint Infection
PLVI: Psoas:Lumbar Vertebrae Index
PROM: Patient Reported Outcome Measure
ROBINS‐I: Risk of Bias in Non‐randomised Studies of Interventions
SF‐MNA: Short‐form Mini Nutritional Assessment
SNAQ: Short Nutritional Assessment Questionnaire
SSI: surgical site infection
THA: total hip arthroplasty
TKA: total knee arthroplasty
UK: United Kingdom
UKA: unicompartmental knee arthroplasty
VAS: Visual Analogue Score
VMS: Veiligheids Management System Frailty Score
WOMAC: Western Ontario and McMaster University Osteoarthritis Index

## INTRODUCTION

Malnutrition affects approximately 5% of the English population with an associated cost to the healthcare system of £22.6 billion per annum, £15.7 billion of which is spent in secondary care [20]. Female sex, age 65 years or older, emergency presentations and winter admission are factors associated with increased risk of malnutrition, mirroring the characteristics of arthroplasty patients [50, 57]. Systematic review has linked malnutrition with increased morbidity, mortality and length of hospital stay following arthroplasty [7]. Given fifty percent of malnutrition‐associated costs can be attributed to patients aged 65 years and older, the aging global and UK population represent a growing challenge for arthroplasty surgeons [15, 53].

Prior systematic review into the effect of nutritional disorder following joint arthroplasty is limited by the absence of recommended, nutrition specific terminology in the search strategy and heterogenous use of diagnostic criteria that differ from those outlined by the European Society of Clinical Nutrition and Metabolism (ESPEN) [7, 9]. Furthermore, the included definitions of nutritional disorder focussed on malnutrition whereas frailty, sarcopenia, micronutrient deficiency, refeeding syndrome and overweight/obesity‐associated disorders represent other nutritional abnormalities that may have adverse effect following joint arthroplasty (Figure 1 and Table 1) [9]. Given the reported prevalence of nutritional disorder: 0.9%–71.7% in primary arthroplasty, 1.33%–47.5% in revision arthroplasty and 4.5%–60% in arthroplasty following hip fracture, it is important to determine their effect following hip and knee arthroplasty [39].

**Figure 1:**
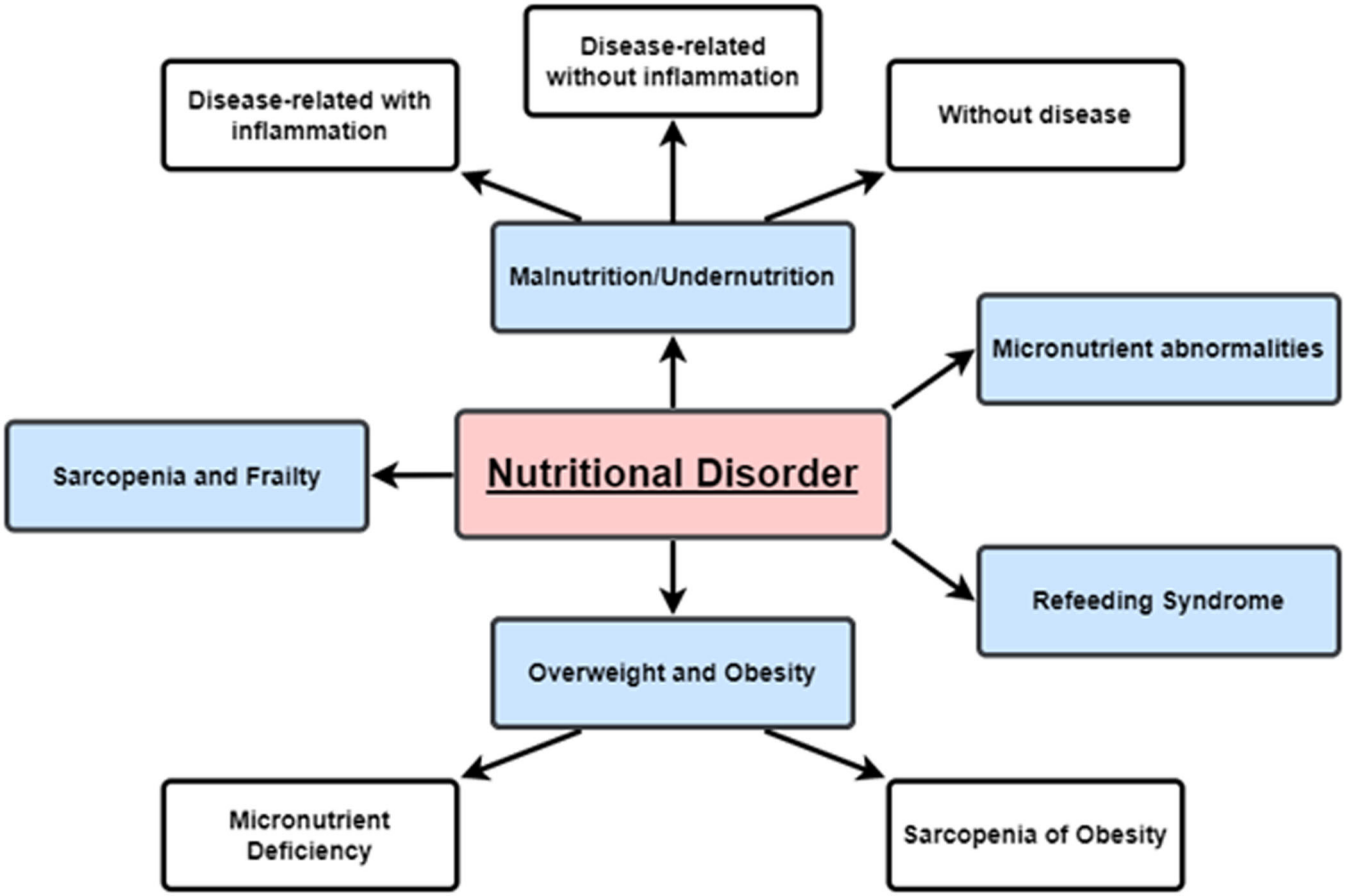
European Society of Parenteral and Enteral Nutrition (ESPEN) spectrum of nutritional disorder [9].

**Table 1:** ESPEN definitions of nutritional disorder [7, 9, 71].

Term	ESPEN definition(s) and explanation
Malnutrition	ESPEN:
	BMI<18.5 kg/m^2^ OR weight loss (>5% in < 3 months or >10% habitually) AND either a reduction in age‐dependent BMI (<20 kg/m^2^ for patients < 70years and < 22 kg/m^2^ if ≥70years) or gender‐dependent fat free mass index (<15 kg/m^2^ for women and <17 kg/m^2^ for men)
	ASPEN:
	2≤ of the following: low energy intake, weight loss, loss of muscle mass, loss of subcutaneous fat, fluid accumulation and/or reduced hand‐grip strength
Disease‐related malnutrition (DRM) with inflammation	A catabolic state associated with disease that has biochemical evidence of acute or chronic inflammation, leading to anorexia, reduced food intake, weight loss and muscle catabolism.
Disease‐related malnutrition (DRM) without inflammation	A catabolic state associated with disease that has no biochemical indices of present or recurrent inflammation i.e. any disease state that interferes with the intake or absorption of nutrients. Some conditions such as IBD may have components of both DRM with inflammation and without inflammation
Malnutrition without disease	Malnutrition related to either starvation, socioeconomic or psychological factors i.e. in the absence of any physical health conditions that may alter intake or absorption or induce an inflammatory response
Sarcopenia	Progressive and generalised loss of skeletal muscle mass, strength and function with a consequent risk of adverse outcomes. This can be primary due to ageing (a precursor to frailty) or secondary to malnutrition
Frailty	Whilst there is no unifying consensus, it is generally accepted that frailty is a state of vulnerability and non‐resilience with limited reserve capacity in major organ systems.
	Frailty has a multi‐factorial aetiology: ageing with altered hormonal and neurotransmitter balance affecting hunger and satiety, DRM with and without inflammation due to associated comorbidities and malnutrition without disease state due to isolation, poor functionality and psychological disturbances
Sarcopenia of obesity	Reduced fat free muscle mass in individuals with high BMI secondary to inflammation and disuse atrophy
Micronutrient abnormalities	Deficiency of one or more micronutrients secondary to poor intake, poor absorption or excess consumption of other food stuffs
Refeeding syndrome (RFS)	Severe disruption in electrolyte or fluid balance that is precipitated in malnourished subjects when feeding (oral, enteral or parenteral nutrition) is begun too aggressively after a period of inadequate nutrition.
	Risk factors: BMI<16 kg/m^2^; unintentional weight loss >15% in 3–6 months; little or no intake for >10 days; or low potassium, phosphate and magnesium before feeding.
	Pathophysiology: The release of insulin and other metabolic changes associated with eating causes a rapid decline in in plasma ion concentrations and changes in fluid balance.

Abbreviations: ASPEN, American Society for Parenteral and Enteral Nutrition; BMI, body mass index; ESPEN, European Society of Parenteral and Enteral Nutrition.

The aim of this article is to address limitations of prior systematic review, using ESPEN terminology and diagnostic criteria to test the hypothesis that different nutritional disorders have adverse effect on clinical outcomes and healthcare costs following hip and knee arthroplasty [9].

## METHODS

### Literature search

The study was registered with PROSPERO (ID: CRD42023360517). On the 6 September 2022, electronic databases AMED, CENTRAL, EMABASE, MEDLINE, Scopus and Web of Science were searched (Supporting Information: Additional File 1). The search was updated in March 2024. The references of seminal systematic reviews and meta‐analyses were reviewed to check for any additional papers that may satisfy inclusion criteria. A data restriction of papers later than the year 2000 was specified to better reflect current day practice.

### Study selection

Study screening by title, abstract and full text was performed independently by two reviewers (CL and HSP), any disagreements were settled by a senior author (TH).

Randomised controlled trials, registry studies, cohort studies and case series that define nutritional disorder or risk of nutritional disorder were included if they reported the effect of said disorder on an outcome of interest following hip and/or knee arthroplasty. Reviews, case reports and conference abstracts were excluded.

Studies that had an ambiguous definition(s) of nutritional disorder, lacked a comparator without nutritional disorder, reported nutritional disorder in the postoperative period only or did not have an English, full‐text available were excluded.

Obesity and diabetes are co‐morbidities associated with both nutritional disorder and inflammation [8, 9]. To avoid confounding, overweight, obese and diabetic patients were only included if additional criteria were used to diagnose a concurrent nutritional disorder [8, 9]. A body mass index (BMI) < 18.5 kg/m^2^ meets the ESPEN criteria for malnutrition; in this instance, BMI alone was sufficient for inclusion into this study [8, 9].

Hip fracture studies were excluded if they contained, or failed to exclude, pathological fractures. Studies that contained subtrochanteric fractures were also excluded given their association with severe hip osteoarthritis and malignancy, conditions that would confound the hip fracture cohort owing to differences in aetiology, management and prognosis. It was not anticipated that fracture pattern (displaced vs undisplaced intracapsular or intracapsular vs extracapsular) would confound nutritional status when pathological and subtrochanteric fractures were excluded. For this reason, hip fracture studies that contained dynamic hip screw or intramedullary nailing, in addition to arthroplasty, were included unless it could be identified that they contained <50 arthroplasty cases. Studies which included only fixation techniques were excluded.

The outcomes of interest for this study were: (1) mortality, (2) complication rate, (3) re‐operation rate, (4) revision rate, (5) rate of prosthetic joint or surgical site infection (PJI/SSI), (6) patient reported outcome measure (PROM) data, (7) length of hospital stay (LOHS) and (8) economic analyses.

### Data extraction

Data extractions were performed independently and in duplicate using a templated document to ensure consistency (CL and HSP), disagreements were resolved by consultation with a senior author (TH).

### Assessment of methodological quality

As outlined in the Cochrane Handbook for Systematic reviews and interventions, risk of bias was assessed using the risk of bias in non‐randomised studies of interventions (ROBINS‐I) tool [45, 63].

### Statistical analyses

Our protocol planned for pooled analyses with a random‐effects model but heterogeneity in study demographics, definitions of nutritional disorder and reported outcome measures prevented this.

## RESULTS

Two‐thousand, eight‐hundred and eighty‐three unique articles were identified, from which 184 full‐text articles were accessed for eligibility. Fifty‐eight studies met the inclusion criteria and were included in the systematic review (Figure 2) [1, 2, 3, 4, 5, 6, 10, 11, 12, 13, 16, 17, 18, 19, 21, 22, 24, 25, 26, 27, 28, 29, 30, 31, 32, 33, 34, 35, 37, 38, 40, 41, 42, 43, 44, 46, 47, 48, 49, 51, 52, 55, 59, 60, 61, 62, 64, 65, 66, 67, 68, 70, 72, 73, 74, 75, 76, 77]. The total number of patients included in this study was 2,076,289. However, the study dates overlap between 12 National Surgical Quality Improvement Program (NSQIP) studies (10 in primary arthroplasty and two in hip fracture cohorts) and between two studies from the same centre in Germany [4, 6, 16, 19, 28, 29, 31, 44, 47, 48, 49, 59, 70, 72]. The 10 NSQIP primary arthroplasty studies also overlap with one National Readmission Database study [4, 6, 16, 19, 31, 38, 44, 47, 48, 49, 59, 70, 72]. Duplication of patient entry into this systematic review cannot, therefore, be excluded. The study demographics can be found in Supporting Information: Additional File 2.

**Figure 2:**
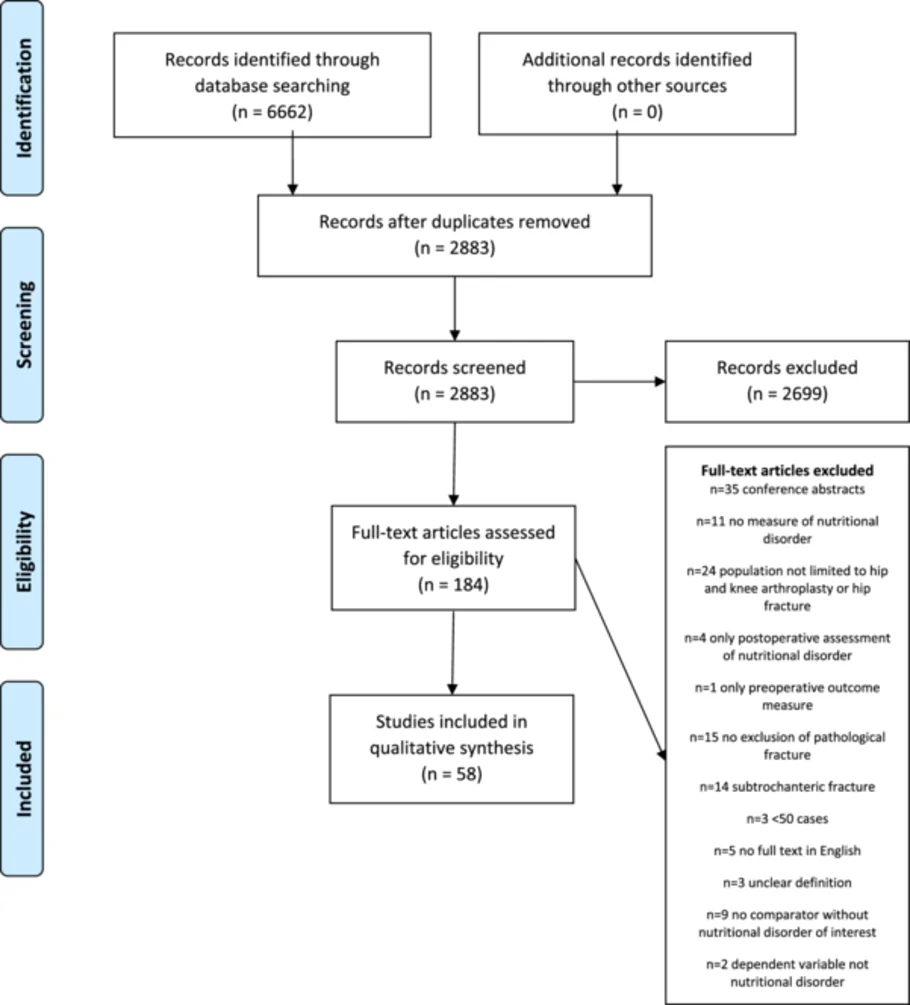
PRISMA flow diagram for inclusion and exclusion [23, 45].

### Methodological quality

Thirty‐four studies had a moderate risk of bias and 24 a serious risk (Figures 3 and 4). A moderate risk of confounding was present if studies used regression to account for differences in patient characteristics, serious risk was attributed to studies that did not use such methods or failed to report baseline characteristics [29, 34, 35]. All retrospective studies had a moderate risk of selection bias due to the exclusion of patients without diagnostic or outcome data. Moderate selection biases were also present in Bohl et al., which relied on coding to identify the indication for revision, and in both Cook et al. papers due to the requirement for patients to be registered with a general practice that signs up to the clinical practice research datalink (CPRD) [5, 11, 12]. As a case‐control study, Babu et al. had moderate selection bias in the choosing of a control group [1]. Seventeen studies had serious selection bias due to the exclusion of patients with comorbidity affecting nutritional status [11, 21, 24, 27, 29, 30, 32, 33, 41, 42, 43, 44, 46, 47, 64, 66, 70]. Tram et al. excluded deceased patients and Yi et al. those with early revision, also leading to serious selection biases [67, 75]. Two studies used the median value to define sarcopenia following computed tomography (CT) scan, introducing moderate classification bias [1, 2]. Two studies performed receiver operating characteristic analyses to determine a cut‐off value that subsequently defined nutritional disorder, before demonstrating said disorder had significant effect; this introduced a serious risk of bias due to classification of intervention and reporting [24, 41]. Tan et al. had severe bias due to deviation given the administration of albumin to patients with serum levels <3 g/L at Day 3 post‐op [64]. Most of the studies had moderate bias due to missing data secondary to the retrospective nature of the studies. Moderate bias due to measurement of outcomes were present in most studies as outcomes such as SSI, pneumonia and wound complication are not objective measures. Two studies had a low risk of reporting bias due to publicly available protocols [28, 29].

**Figure 3:**
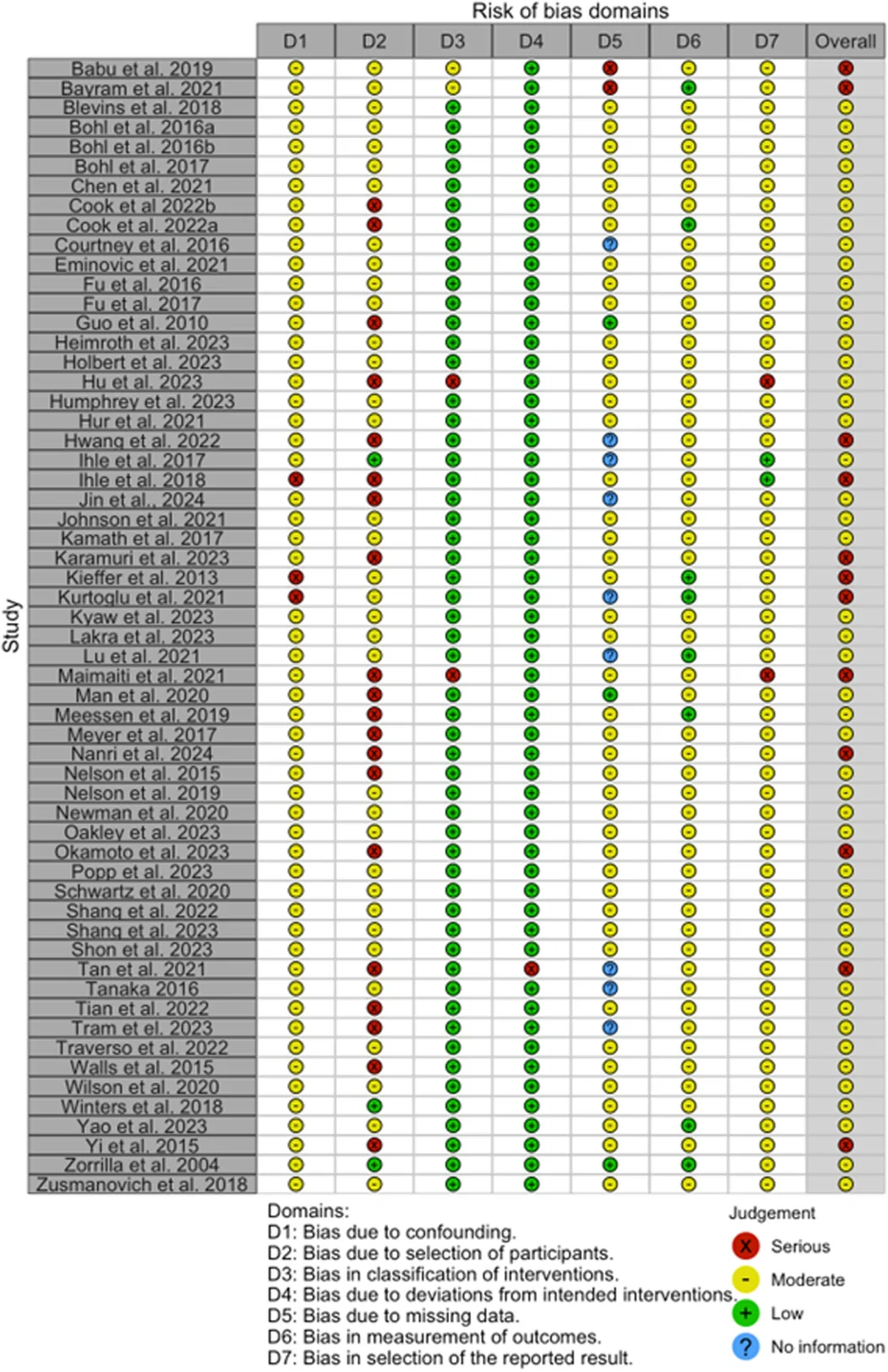
ROBINS‐I assessment of non‐randomised studies [63].

**Figure 4:**
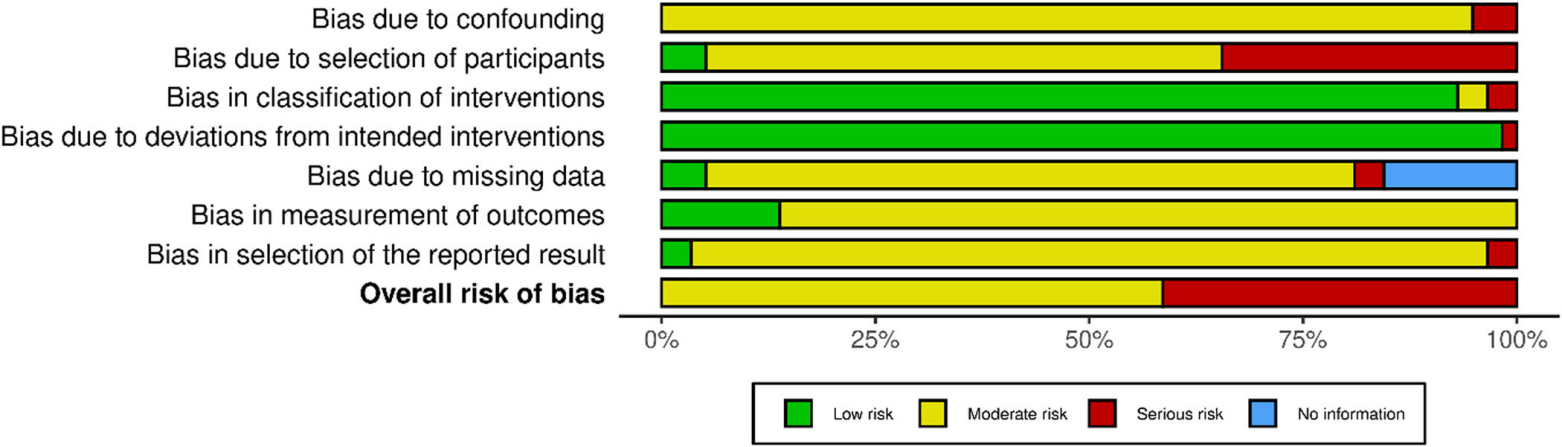
ROBINS‐I summary [63].

### ESPEN criteria (Table 2)

**Table 2:** Outcomes for nutritional disorders that satisfy ESPEN recommended diagnostic criteria ±1.96 standard deviation from mean.

Population	Outcome	Definition nutritional disorder	*n*	Disorder	Comparator	Effect size(s)/*p*‐value	Other data reported	References
Primary total hip arthroplasty	Mortality	Underweight (BMI < 18.5 kg/m2)	840	0%	0%	/	/	[77]
		Electronic Frailty Index				30‐day		[11]
		Mild Frailty (0.12 <–≤ 0.24)				HR 0.87 [0.66‐1.15]		
		Moderate Frailty (0.24 <–≤ 0.36)				HR 1.73 [1.26‐2.38]		
		Severe Frailty (>0.36)	103,563	/	/	HR 2.85 [1.84‐4.39]	/	
		Modified Frailty Index 2≤	105,997	0.3%	0.10%	30‐day	/	[72]
						OR 2.02 [1.37‐2.96] *p* < 0.001		
	Complication rate	Underweight (BMI < 18.5 kg/m^2^)	840	/	/	/	<18.5 kg/m^2^ vs > 18.5 kg/m^2^:	[77]
							Operation Time (min) 96.0 vs 89.6. p = 0.023	
							No significant difference in rates of pulmonary embolism, deep vein thrombosis, pneumonia, urinary tract infection, stroke, cardiac arrest, myocardial infarction, transfusion requirement or wound disruption	
		Nutrition Risk Screening 2002 3≤	89	0.60 ± 0.52%	0.36 ± 0.48%	*p*>0.05	/	[28]
		Electronic Frailty Index	42,512	/	/	/	Oxford pain sub‐scores adjusted for age, sex, deprivation and pre‐op score. β‐coefficient (95% CI)	[12]
		Mild Frailty (0.12 <–≤ 0.24)					Mild ‐1.16 (‐1.26, ‐1.06)	
		Moderate Frailty (0.24 <–≤ 0.36)					Moderate ‐2.25 (‐2.39, ‐2.11)	
		Severe Frailty (>0.36)					Severe ‐3.13 (‐3.37, ‐2.90)	
		Modified Frailty Index 2≤	105,997	Minor 3.1%	Minor 1.6%	Minor: OR 1.67 [1.5–1.87] *p* < 0.001	/	[72]
				Major 3.1%	Major 1.6%	Major: OR 1.68 [1.5–1.88] *p* < 0.001		
		SARC‐CalF 11≤	356	/	/	Ability of the patient to walk more than 50 m independently, with or without a walking aid.	/	[46]
						HR 0.55 (0.39–0.78) *p* < 0.001		
		Calf circumference (cm):	356	/	/	Ability of the patient to walk more than 50 m independently, with or without a walking aid.	/	[46]
		Female <33						
		Male <34				HR 0.57 (0.41–0.80) *p* = 0.001		
	Re‐operation Rate	Underweight (BMI < 18.5 kg/m2)	840	1.3%	0.9%	*p* = 0.729	/	[77]
		Groningen Frailty Indicator 4≤	805	6.4%	2.4%	*p* = 0.005	/	[43]
	No data available for revision rate							
	PJI/SSI Rate	Underweight (BMI < 18.5 kg/m^2^)	840	Superficial SSI: 0.3%	Superficial SSI: 0.4%	*p* = 0.707	/	[77]
				Deep SSI: 0%	*p* = 1			
		Modified Frailty Index 2 ≤ :	105,997	Superficial SSI	Superficial SSI 0.5%	Multivariate Analysis	/	[72]
				0.9%		Superficial SSI:		
				Deep SSI		OR 1.4 [95% CI, 1.19–1.79] *p* < 0.001		
				0.4%	Deep SSI 0.2%	Deep SSI:		
						OR 1.77 [95% CI, 1.31–2.38] *p* < 0.001		
	PROM Data	Electronic Frailty Index	42,512	/	/	/	Oxford hip score adjusted for age, sex, deprivation and pre‐op score. β‐coefficient (95% CI)	[12]
		Mild Frailty (0.12 <–≤ 0.24)					Mild ‐2.12 (‐2.29, ‐1.94)	
		Moderate Frailty (0.24 <–≤ 0.36)					Moderate ‐4.28 (‐4.52, ‐4.03)	
		Severe Frailty (>0.36)					Severe ‐6.37 (‐6.79, ‐5.95)	
		Groningen Frailty Indicator 4≤	805	/	/	/	Non‐frail patients had a significantly greater improvement in HOOS sports sub‐scales from baseline to 12‐months post‐op: 49.4 vs 42.6, p = 0.002	[43]
		Psoas muscle index (cm^2^/m^2^):	205	Achieved MCID:	Achieved MCID:	Achieved MCID:	/	[52]
		Female <3.92		EQ. 5D: 45.4%	EQ. 5D: 68.5%	EQ. 5D: *p* < 0.001		
		Male <6.36		VAS hip pain: 87.6%	VAS hip pain: 92.6%	VAS‐hip pain: *p* = 0.232		
				VAS low back pain: 62.9%	VAS low back pain: 85.2%	VAS‐low back pain: *p* < 0.001		
				HOOS: 77.3%	HOOS: 90.7%	HOOS: *p* = 0.008		
	LOHS (days)	Underweight (BMI < 18.5 kg/m2)	840	3.4	3.1	*p* = 0.048	/	[77]
		Nutrition Risk Screening 2002 3≤	/	14.5 ± 4.4	12.1 ± 2.	*p* > 0.05	/	[28]
		Modified Frailty Index 2 ≤ :	105,997	>2 days	>2 days	Multivariate Analysis	/	[72]
				54.4%	40.4%	OR 1.59 [1.54–1.65] *p* < 0.001		
	No data available for economic analysis							
Primary total knee arthroplasty	Mortality	Electronic Frailty Index:	125,367	/	/	30‐day	/	[11]
		Mild Frailty (0.12 <–≤ 0.24)				HR 1.31 [0.97–1.77]		
		Moderate Frailty (0.24 <–≤ 0.36)				HR 1.73 [1.22–2.46]		
		Severe Frailty (>0.36)				HR 2.14 [1.29–3.53]		
		Modified Frailty Index 2 ≤ :	179,702	0.2%	0.1%	30‐day	/	[59]
						OR 2.35 [1.75–3.16] *p* < 0.001		
		Frailty Index: non‐frail ( < 0.11), vulnerable (0.11–0.2) and frail (0.2 < )	435	1 year	1 year	/	/	[33]
				Vulnerable 0.96%	0%			
				Frail 4.08%				
	Complication Rate	Nutrition Risk Screening 2002 3≤	/	0.24 ± 0.43%	0.20 ± 0.42%	*p*>0.05	/	[28]
		Electronic Frailty Index:	49,208	/	/	/	Oxford pain sub‐scores adjusted for age, sex, deprivation and pre‐op score. β‐coefficient (95% CI)	[12]
		Mild Frailty (0.12 <–≤ 0.24)						
		Moderate Frailty (0.24 <–≤ 0.36)					Mild ‐1.11 (‐1.22, ‐0.99)	
		Severe Frailty (>0.36)					Moderate ‐2.14 (‐2.28, ‐2.00)	
							Severe ‐3.31 (‐3.55, ‐3.06)	
		Modified Frailty Index 2 ≤ :	179,702	Minor 4.3%	Minor 1.5%	Minor: OR 1.44 [1.32–1.57] *p* < 0.001	/	[59]
				Major 5.7%	Major 2.1%	Major: OR 1.37 [1.27–1.48] *p* < 0.001		
		Hospital Frailty Risk Score 5≤	882,479	30‐day readmission: 5.3%	30‐day readmission: 2.6%	30‐day readmission: OR: 1.89, *p* = 0.001	/	[38]
				Surgical complication 0.6% (periprosthetic fracture, dislocation, wound healing disorder and joint infection)	Surgical complication 0.3% (periprosthetic fracture, dislocation, wound healing disorder and joint infection)			
				Medical complications 3.4% (ACS, Decompensated HF, Arrhythmia, Pneumonia and AKI)	Medical complications 1.3% (ACS, Decompensated HF, Arrhythmia, Pneumonia and AKI)	Surgical complication: *p* < 0.01		
						Medical complications: *p* < 0.01		
						Other complications: *p* < 0.01		
				Other complications 0.9% (Thrombosis, PE, Syncope, CVA and Delirium)	Other complications 0.5% (Thrombosis, PE, Syncope, CVA and Delirium)			
		Frailty Index: non‐frail ( < 0.11), vulnerable (0.11–0.2) and frail (0.2 < )	435	/	/	Postoperative Complications	/	[33]
						Frail: HR 1.62 (1.15–1.81)		
						Vulnerable: HR 1.26 (1.10–1.45)		
						Re‐admission within 1 year		
						Frail: HR 3.12 (1.85–3.90)		
						Vulnerable: HR 0.96 (0.91–1.12)		
		Comprehensive Geriatric Assessment‐Frailty Index (CGA‐FI) 0.25<	164	CPSP 43.5%	CPSP 7.1%	*p* < 0.001 for both	AUC for frailty:	[30]
							CPSP 0.728 (0.651 – 0.805)	
				APSP 44.4%	APSP 5.4%		APSP 0.756 (0.673 – 0.839)	
							p < 0.001 for both	
		Appendicular Skeletal Muscle Index (kg/m^2^)						
		Female <5.4						
		Male <7.0	445	Cardiovascular: 2.4%	Cardiovascular: 1.7%	Cardiovascular: *p* = 0.551	/	[62]
				Pulmonary: 2.4%	Pulmonary: 1.2%	Pulmonary: *p* = 0.450		
				Hepatic: 9.5%	Hepatic: 9.9%	Hepatic: *p* = 0.934		
				Urologic: 19.0%	Urologic: 19.6%	Urologic: *p* = 0.931		
				Cerebral: 2.4%	Cerebral: 1.5%	Cerebral: *p* = 0.503		
				Delirium: 16.7%	Delirium: 9.7%	Delirium: *p* = 0.179		
				Blood transfusion: 28.6%	Blood transfusion: 4.7%	Blood transfusion: *p* < 0.001		
				DVT (distal): 9.5%	DVT (proximal): 1.7%	DVT (proximal):		
				Infection: 4.8%	DVT (distal): 9.4%	*p* = 0.389		
						DVT (distal): *p* = 0.984		
		Appendicular Skeletal Muscle Index (kg/m^2^)	70	Transfusion: 28.6%	Transfusion: 5.7%	Transfusion: OR 6.6 (1.57‐45.4) *p* = 0.021	ROC analyses for post‐op transfusion with cut‐off <5.6 kg/m^2^	[27]
		Female <5.7		AKI: 2.9%	AKI: 17.1%	AKI: OR 0.14 (0.01–0.90) *p* = 0.079	AUC 0.797 (0.633–0.96)	
		Male <7.0		Delirium: 11.4%	Delirium: 5.7%	Delirium: OR 2.13 (0.39‐16.1) *p* = 0.4	Sensitivity 66.7%,	
							Specificity 86.2%	
		SARC‐CalF 11≤	243	/	/	Ability of the patient to walk more than 50 m independently, with or without a walking aid.	/	[46]
						HR 1.22 (0.84‐1.78) *p* = 0.297		
		Calf circumference (cm):	243	/	/	Ability of the patient to walk more than 50 m independently, with or without a walking aid.	/	[46]
		Female <33						
		Male <34				HR 0.87 (0.59‐1.28) *p* = 0.489		
	Re‐operation Rate	Modified Frailty Index 2 ≤ :	179,702	1.5%	1%	OR 1.35 [1.21–1.5] *p* < 0.001	/	[59]
		Groningen Frailty Indicator 4≤	640	3.7%	2.1%	*p* = 0.278	/	[43]
		Hospital Frailty Risk Score 5≤	882,479	30‐day reoperation	30‐day reoperation	*p* < 0.001	/	[38]
				0.6%	0.3%			
	Revision rate	Frailty Index: non‐frail (< 0.11), vulnerable (0.11–0.2) and frail (0.2<)	435	Frail 4%	0.56%	Frail *p* = 0.12	/	[33]
				Vulnerable 0.4%		Vulnerable *p* = 1		
	PJI/SSI Rate	Modified Frailty Index 2 ≤ :	179,702	Superficial SSI 1.1%	Superficial SSI 0.5%	Multivariate analysis	/	[59]
						Superficial SSI:		
				Deep SSI 0.6%	Deep SSI 0.1%	OR 1.12 [95% CI, 0.95–1.32] *p* = 0.194		
						Deep SSI:		
						OR 1.60 [95% CI, 1.16–2.21] *p* = 0.005		
		Frailty Index: non‐frail ( < 0.11), vulnerable (0.11–0.2) and frail (0.2<)	435	PJI	PJI	Frail *p* = 0.039	/	[33]
				Frail 2.04%				
				Vulnerable 0.96%	0.56%	Vulnerable *p* = 1		
		Hospital Frailty Risk Score 5≤	882,479	PJI 0.4%	PJI 0.3%	*p* < 0.001	/	[38]
	PROM Data	Electronic Frailty Index:	49,208	/	/	/	Oxford knee score adjusted for age, sex, deprivation and pre‐op score. β‐coefficient (95% CI)	[12]
		Mild Frailty (0.12 <–≤ 0.24)						
		Moderate Frailty (0.24 <–≤ 0.36)					Mild ‐1.81 (‐1.99, ‐1.62)	
		Severe Frailty (>0.36)					Moderate ‐3.61 (‐3.85, ‐3.80)	
							Severe ‐5.74 (‐6.14, ‐5.34)	
		Groningen Frailty Indicator 4≤	640	/	/	/	Non‐frail patients had a significantly greater improvement in HOOS sports and QoL sub‐scales from baseline to 12‐months post‐op:	[43]
							Sports 36.4 vs 27.9, p < 0.001 QoL 19.1 vs 16.1, p = 0.02	
							Only the Sports subclass of KOOS reached the minimal clinically important difference (MCID) of 10.	
		Skeletal Muscle Index (cm^2^/m^2^):	140	/	/	OR Sarcopenia achieving MCID	/	[25]
		Females <39				KOOS JR OR 0.31 (0.10–0.97) *p* = 0.04		
		Males <53				PROMIS‐PF‐SF10a OR 0.32 (0.12–0.85) *p* = 0.02		
		Appendicular Skeletal Muscle Index (kg/m^2^)	445	KOOS Pain:	KOOS Pain:	KOOS Pain:	/	[62]
		Female <5.4		Preop: 43.8 ± 8.1	Preop: 44.3 ± 8.5	Preop: 0.738		
		Male <7.0		3 months: 80.7 ± 6.0	3 months: 81.0 ± 7.9	3 months: 0.681		
				6 months: 82.1 ± 6.1	6 months: 82.0 ± 6.5	6 months: 0.731		
				12 months: 84.1 ± 4.9	12 months: 84.2 ± 5.1	12 months: 0.542		
						KOOS Symptom:		
						Preop: 0.814		
				KOOS Symptom:	KOOS Symptom:	3 months: 0.047		
				Preop: 47.2 ± 9.2	Preop: 46.8 ± 8.1	6 months: 0.026		
				3 months: 60.1 ± 6.2	3 months: 65.4 ± 6.1	12 months: 0.061		
				6 months: 69.4 ± 4.2	6 months: 76.1 ± 4.9			
				12 months: 71.4 ± 2.1	12 months: 78.1 ± 3.9	KOOS ADL:		
				KOOS ADL:	KOOS ADL:	Preop: 0.713		
				Preop: 42.1 ± 6.8	Preop: 42.8 ± 5.3	3 months: <0.001		
				3 months: 65.1 ± 8.2	3 months: 75.4 ± 6.1	6 months: <0.001		
				6 months: 73.8 ± 2.2	6 months: 80.1 ± 2.7	12 months: 0.106		
				12 months: 81.8 ± 2.2	12 months: 83.1 ± 1.7	KOOS Sport/Rec:		
				KOOS Sport/Rec:	KOOS Sport/Rec:			
				Preop: 33.2 ± 5.8	Preop: 32.9 ± 3.9	Preop: 0.523		
				3 months: 45.1 ± 8.2	3 months: 55.4 ± 5.1	3 months: <0.001		
						6 months: <0.001		
				6 months: 67.8 ± 3.2	6 months: 77.1 ± 4.7	12 months: 0.720		
				12 months: 75.1 ± 3.7	12 months: 77.7 ± 4.2	KOOS QOL:		
						Preop: 0.832		
				KOOS QOL:	KOOS QOL:			
						3 months: <0.001		
						6 months: <0.001		
						12 months: 0.638		
						WOMAC Pain:		
						Preop: 0.614		
				Preop: 40.1 ± 7.1	Preop: 40.7 ± 4.8	3 months: 0.031		
						6 months: 0.017		
				3 months: 72.1 ± 9.1	3 months: 78.7 ± 8.8	12 months: 0.532		
				6 months: 78.9 ± 5.0	6 months: 84.1 ± 3.2			
						WOMAC Stiffness:		
				12 months: 82.9 ± 1.9	12 months: 82.1 ± 2.3	Preop: 0.729		
						3 months: 0.032		
						6 months: 0.017		
				WOMAC Pain:	WOMAC Pain:	12 months: 0.817		
				Preop: 9.6 ± 7.2	Preop: 9.7 ± 4.1			
						WOMAC Function:		
				PO at 3 months: 6.6 ± 5.2	PO at 3 months: 4.7 ± 4.1	Preop: 0.738		
				PO at 6 months: 3.7 ± 4.3	PO at 6 months: 2.8 ± 2.9	3 months: <0.001		
				PO at 12 months: 2.4 ± 2.3	PO at 12 months: 2.3 ± 2.9	6 months: <0.001		
						12 months: 0.068		
				WOMAC Stiffness:	WOMAC Stiffness:			
				Preop: 5.2 ± 2.1	Preop: 5.6 ± 2.7	WOMAC Total:		
						Preop: 0.828		
				3 months: 3.8 ± 6.1	3 months: 2.9 ± 4.7	3 months: <0.001		
				6 months: 3.3 ± 1.6	6 months: 2.1 ± 1.9	6 months: <0.001		
				12 months: 2.3 ± 1.6	12 months: 2.2 ± 1.9	12 months: 0.272		
				WOMAC Function:	WOMAC Function:			
				Preop: 34.1 ± 5.5	Preop: 33.8 ± 6.1			
				3 months: 24.1 ± 9.5	3 months: 15.1 ± 5.1			
				6 months: 19.2 ± 6.9	6 months: 8.7 ± 2.3			
				12 months: 9.2 ± 2.9	12 months: 7.7 ± 2.3			
				WOMAC Total:	WOMAC Total:			
				Preop: 48.9 ± 7.1	Preop: 49.1 ± 4.6			
				3 months: 34.5 ± 6.8	3 months: 22.7 ± 4.9			
				6 months: 26.2 ± 5.8	6 months: 13.6 ± 2.4			
				12 months: 11.2 ± 2.5	12 months: 12.2 ± 2.3			
	LOHS (days)	Nutrition Risk Screening 2002 3≤	/	13.2 ± 6.1	11.7 ± 2.3	*p* > 0.05	/	[28]
		Modified Frailty Index 2 ≤ :	179,702	>2 days	>2 days	Multivariate analysis	/	[59]
				66.7%	57.6%	OR 1.38 (1.35–1.42) *p* < 0.001		
		Hospital Frailty Risk Score 5≤	882,479	4.1 days	2.2 days	IRR: 1.43 (1.43–1.44) *p* = 0.001	/	[38]
	Economic Analysis	Hospital Frailty Risk Score 5≤	882,479	$19,841	$6104	IRR: 1.16 (1.16–1.17) *p* = 0.001	/	[38]
All‐form primary hip arthroplasty (total and hemiarthroplasty)	No data available for any outcome of interest							
Unicompartmental knee arthroplasty	No data available for any outcome of interest							
Primary total hip and knee arthroplasty (combined cohorts)	No mortality data reported							
	Complication Rate	Nutrition Risk Screening 2002 3≤	649	**Ihle 2018**	**Ihle 2018**	**Ihle 2018**	/	[29, 60]
						*p* = 0.37		
				40.6%	32.7%	**Shang 2022**		
						Short‐term OR 7.7 [1.99–29.836]		
						Long‐term OR 6.115 [1.6–23.31]		
		Short‐form Mini Nutritional Assessment <7	204	SF‐MNA < 7 vs. 7≤			/	[29]
				On admission: 39.4% vs. 31.8% *p* = 0.25				
				6‐months post‐surgery: 12.8% vs. 7.6% *p* = 0.28				
		Mini Nutritional Assessment ≤11	204	MNA ≤ 11 vs. 11<			/	[29]
				On admission: 43.2% vs. 29.9% *p* = 0.03				
				6‐months post‐surgery: 15.1% vs. 6.6% *p* = 0.06				
	No data available for re‐operation or revision rate							
	PJI/SSI Rate	Body Mass Index <18.5 kg/m^2^	30,863	0.01%	0.01%	*p* = 0.758	/	[3]
		PLVI < 0.842	99	44.9%	16%	OR 0.5 (0.263–0.966) *p* = 0.039	/	[1]
	No data available for PROMs							
	LOHS (days)	PLVI < 0.842.	99	3.77	3.54	*p* = 0.975	/	[1]
	No data available for economic analysis							
Revision hip arthroplasty	No data available for mortality							
	Complication Rate	Hospital Frailty Risk Score 5≤	15,448 Dislocation	**Dislocation**	**Dislocation**	**Dislocation**	/	[67]
			11,062 Mechanical Loosening	Surgical 11.3% (periprosthetic fracture, dislocation, wound healing disorder and joint infection)	Surgical 6.9% (periprosthetic fracture, dislocation, wound healing disorder and joint infection)	Surgical *p* < 0.01		
			9733 Infection	Medical 10.6% (ACS, Decompensated HF, Arrhythmia, Pneumonia and AKI)	Medical 3.8% (ACS, Decompensated HF, Arrhythmia, Pneumonia and AKI)	Medical *p* < 0.01		
				Other 2% (Thrombosis, PE, Syncope, CVA and Delirium)	Other 0.8% (Thrombosis, PE, Syncope, CVA and Delirium)	Other *p* < 0.01		
				**Mechanical loosening**	**Mechanical loosening**	**Mechanical loosening**		
				Surgical 4.1%	Surgical 3%	Surgical *p* = 0.03		
				Medical 7%	Medical 2.4%	Medical *p* < 0.01		
				Other 1.5%	Other 0.6%	Other *p* < 0.01		
				**Infection**	**Infection**	**Infection**		
				Surgical 15.1%	Surgical 10.3%	Surgical *p* < 0.01		
				Medical 11.6%	Medical 5.5%	Medical *p* < 0.01		
				Other 2.6%	Other 1.2%	Other *p* < 0.01		
	Re‐operation Rate ( < 30 days)	Hospital Frailty Risk Score 5≤	15,448 Dislocation	Dislocation 9%	Dislocation 6.1%	Dislocation *p* < 0.01	/	[67]
			11,062 Mechanical Loosening	Mechanical Loosening 3.5%	Mechanical Loosening 2.6%	Mechanical Loosening *p* = 0.03		
			9733 Infection	Infection 7.3%	Infection 5.4%	Infection *p* < 0.01		
	No data available for re‐revision rate							
	PJI/SSI Rate	Hospital Frailty Risk Score 5≤	15,448 Dislocation	**Joint Infection**	**Joint Infection**	Dislocation *p* < 0.01	/	[67]
			11,062 Mechanical Loosening	Dislocation 4.3%	Dislocation 2.9%	Mechanical Loosening *p* = 0.37		
				Mechanical Loosening 2.1%	Mechanical Loosening 1.7%			
			9733 Infection	Infection 13.7%	Infection 9.1%	Infection *p* < 0.01		
	No data available for PROM data, LOHS or Economic Analyses							
Revision knee arthroplasty	No data available for mortality							
	Complication Rate	Hospital Frailty Risk Score 5≤	25,177 Mechanical Loosening	**Mechanical Loosening**	**Mechanical Loosening**	**Mechanical Loosening**	/	[37]
			12,712 Infection	Surgical 1.8% (periprosthetic fracture, dislocation, wound healing disorder and joint infection)	Surgical 1.3% (periprosthetic fracture, dislocation, wound healing disorder and joint infection)	Surgical *p* = 0.02		
			9458 Instability					
				Medical 5% (ACS, Decompensated HF, Arrhythmia, Pneumonia and AKI)	Medical 1.8% (ACS, Decompensated HF, Arrhythmia, Pneumonia and AKI)	Medical *p* < 0.01		
				Other 1.5% (Thrombosis, PE, Syncope, CVA and Delirium)	Other 0.6% (Thrombosis, PE, Syncope, CVA and Delirium)	Other *p* < 0.01		
				**Infection**	**Infection**	**Infection**		
				Surgical 9.6%	Surgical 6.1%	Surgical *p* < 0.01		
				Medical 10.1%	Medical 4.8%	Medical *p* < 0.01		
				Other 1.7%	Other 1.1%	Other *p* < 0.01		
				**Instability**	**Instability**	**Instability**		
				Surgical 3.7%	Surgical 1.6%	Surgical *p* < 0.01		
				Medical 6.1%	Medical 2.2%	Medical *p* < 0.01		
				Other 1.6%	Other 0.7%	Other *p* < 0.01		
	Re‐operation Rate	Hospital Frailty Risk Score 5≤	25,177 Mechanical Loosening	Mechanical Loosening 1.8%	Mechanical Loosening 1.2%	Mechanical Loosening *p* = 0.01	/	[37]
			12,712 Infection	Infection 3.2%	Infection 2.8%	Infection *p* = 0.23		
			9458 Instability	Instability 3.2%	Instability 1.6%	Instability *p* < 0.01		
	No data available for re‐revision rate							
	PJI/SSI Rate	Hospital Frailty Risk Score 5≤	25,177 Mechanical Loosening	Mechanical Loosening 1.4%	Mechanical Loosening 1.1%	Mechanical Loosening *p* = 0.22	/	[37]
			12,712 Infection	Infection 9.2%	Infection 5.8%	Infection *p* < 0.01		
			9458 Instability	Instability 2.4%	Instability 1%	Instability *p* < 0.01		
	No data available for PROM data, LOHS or Economic Analyses							
Revision hip and knee arthroplasty (combined cohorts)	No mortality data reported							
	Complication Rate	Nutrition Risk Screening 2002 3≤	64	**Ihle 2018**	**Ihle 2017**	[28, 29]		
				NRS 3≤ vs. <3:	NRS 3≤ was not associated with increased risk of mechanical complication			
				At admission: 80.0% vs. 49.1%; *p* = 0.03				
		Short‐form Mini Nutritional Assessment <7	42	72.2%	50%	*p* = 0.1	/	[29]
		Mini Nutritional Assessment ≤11	42	70%	50%	*p* = 0.13	/	[29]
	No data available for re‐operation, revision or PJI/SSI rate or PROM outcomes							
	LOHS	Nutrition Risk Screening 2002 3≤	22	15.6 ± 6.8	15.9 ± 6.0	*p* > 0.05	/	[28]
	No data available for economic analysis							
Hip fracture	Mortality	Mini Nutritional Assessment 0–7	1080	/	/	1 Month OR 5.03 p < 0.01	/	[55]
						2 Months OR 7.28 *p* < 0.01		
						6 Months OR 7.44 *p* < 0.01		
						12 Months OR 7.77 *p* < 0.01		
		Malnutrition Universal Screening Tool 2≤	1080	/	/	1 Month OR 3.06 *p* < 0.01	/	[55]
						2 Months OR 3.48 *p* < 0.01		
						6 Months OR 3.40 *p* < 0.01		
						12 Months OR 2.94 *p* < 0.01		
		Nutrition Risk Screening 2002 3≤	1080	/	/	1 Month OR 2.65 *p* < 0.01	/	[55]
						2 Months OR 2.79 *p* < 0.01		
						6 Months OR 2.74 *p* < 0.01		
						12 Months OR 2.67 *p* < 0.01		
		Short Nutritional Assessment Questionnaire 1≤ (risk of malnutrition)	/	/	/	/	33% of patients that died <30 days where frail according to the SNAQ, p = 0.03	[73]
							21% of patients that died overall where frail according to the SNAQ, p = 0.000	
		Graz Malnutrition Screening 3≤	1080	/	/	1 Month OR 2.34 *p* < 0.01	/	[55]
						2 Months OR 2.70 *p* < 0.01		
						6 Months OR 2.96 *p* < 0.01		
						12 Months OR 2.93 *p* < 0.01		
		Groningen Frailty Indicator 4≤	286	58% overall survival	81% overall survival	HR 2.3 (1.2–4.1) *p* = 0.008	79% of patients that died <30 days where frail according to the GFI, p = 0.165	[73]
							45% of patients that died overall where frail according to the GFI, p = 0.001	
		Veiligheids Management System Frailty Score 2≤	286	57% overall survival	80% overall survival	HR 3.5 (2.1–5.7) *p* < 0.001	91% of patients that died <30 days where frail according to the VMS, p = 0.001	[73]
							76% of patients that died overall where frail according to the VMS, p = 0.000	
		Psoas‐Lumbar Vertebrae Index <0.488	304	/	/	/	PLVI lower in deceased patients: mean 0.49 ± 0.15 vs 0.537 ± 0.14, p = 0.006. Higher % low PLVI in deceased patients: 58.4% vs 40%, p = 0.001.	[2]
	Complication Rate (Delayed Wound Healing)	Mini Nutritional Assessment:	207	25%	7%	*p* < 0.05 for both vs. MNA 23.5<	/	[21]
		Malnutrition <17						
		Risk of Malnutrition 17≤ – ≤23.5		28.5%		Area under receive operative characteristic curve for MNA score: 0.7 [95% CI: 0.62–0.78]		
		Serum Zinc <95 μg/dL	97	/	/	OR 1.13 [95% CI, 1.07‐1.21]	Delayed wound healing related to longer LOHS	[76]
						*p* < 0.001, adjusted for albumin levels		
	No data available for re‐operation, revision or PJI/SSI rate							
	PROM Data (Functional Decline)	Short Nutritional Assessment Questionnaire 1≤	286	18%	/	*p* = 0.074	/	[73]
		Groningen Frailty Indicator 4≤	286	71%	/	*p* = 0.063	/	[73]
		Veiligheids Management System Frailty Score 1≤	286	68%	/	*p* = 0.03	/	[73]
	No data available on LOHS or for economic analysis							
Primary and revision hip and knee arthroplasty	No data available for mortality and no reported rates of complication, re‐operation, revision or PJI/SSI							
	PROM Data	Nutrition Risk Screening 2002 3≤	246	/	/	/	NRS 3≤ had no significant effect on any dimension of the SF‐36	[29]
		Short‐form Mini Nutritional Assessment <7	246	/	/	/	SF‐MNA < 7 showed a statistically significant impact on role‐emotional (p = 0.018) and mental health (p = 0.04) and when adjusted for pre‐op scores, a significant effect on bodily pain (p = 0.02) and general health (p = 0.01)	[29]
		Mini Nutritional Assessment ≤11	/	/	/	/	MNA ≤ 11 shows a statistically significant effect on Physical Functioning (p = 0.02), Role Physical (p = 0.016), General Health (p = 0.004), vitality (p = 0.022), Role Emotional (p = 0.006) and Mental Health (p = 0.044). Data not reported when adjusted for pre‐op values	[29]
	LOHS (days)	Nutrition Risk Screening 2002 3≤	246	14.5 ± 5.3	12.5 ± 3.2	*p* = 0.006	/	[29]
		Short‐form Mini Nutritional Assessment <7	246	13.7 ± 3.9	12.4 ± 3.5	*p* = 0.001	/	[29]
		Mini Nutritional Assessment ≤11	246	13.9 ± 4.3	12.3 ± 3.3	*p* = 0.0001	/	[29]
	No data available for economic analysis							

Abbreviations: [], 95% confidence interval; AKI, acute kidney injury; APSP, acute post‐surgical pain; CPSP, chronic post‐surgical pain; DVT, deep vein thrombosis; EQ5D, EuroQol 5‐Dimension; ESPEN, European Society of Parenteral and Enteral Nutrition; HOOS, Hip Osteoarthritis Outcome Score; HR, hazard ratio; IRR, incidence rate ratio; KOOS, Knee Osteoarthritis Outcome Score; LOHS, length of hospital stay; MCID, minimally clinically important difference; OR, odds ratio; PJI, prosthetic joint infection; PROM, patient reported outcome measure; SSI, surgical site infection; VAS, Visual Analogue Score; WOMAC, Western Ontario and McMaster University Osteoarthritis Index.

Twenty‐six included studies reported effect of nutritional disorder following hip and/or knee arthroplasty when defined using ESPEN recommended criteria [1, 2, 3, 11, 12, 21, 25, 27, 28, 29, 30, 33, 37, 38, 43, 46, 52, 55, 59, 60, 62, 67, 72, 73, 76, 77]. Eight studies reported effect in primary THA [11, 12, 28, 43, 46, 52, 72, 77], twelve in primary TKA [11, 12, 25, 27, 28, 30, 33, 38, 43, 46, 59, 62], four in combined primary THA and TKA [1, 3, 29, 60], one in revision hip [67], one in revision knee [37], two in combined revision hip and knee arthroplasty [28, 29], five in hip fracture [2, 21, 55, 73, 76] and one in combined primary and revision, hip and knee arthroplasty [29]. No included study reported the effect of nutritional disorder as per ESPEN criteria following all‐form primary hip arthroplasty (total and hemiarthroplasty) or UKA.

#### At‐risk of malnutrition

Those at‐risk of malnutrition according to the Nutritional Risk Screening (NRS) 2002 (≤3) had increased rates of short and long‐term complication in the largest study reporting effect in a primary hip and knee arthroplasty cohort [60]. Smaller studies from a single centre show the same effect with respect to complication rate and LOHS, but differences did not reach statistical significance [28, 29]. In the revision hip and knee cohort from this centre, a NRS 2002 score of ≤3 increased likelihood of complication (80% vs. 49.9%, *p* = 0.03) and when primary and revision cohorts where combined, likelihood of increased LOHS (14.5 ± 5.3 vs. 12.5 ± 3.2 days, *p* = 0.006) [29].

Following arthroplasty for hip fracture, being at‐risk of malnutrition as per the NRS 2002, Graz Malnutrition Screening or Short Nutritional Assessment Questionnaire (SNAQ) increased mortality at 1‐month and overall during respective follow‐up periods [55, 73]. Increased complication rate was associated with a mini nutritional assessment (MNA) score of ≤17 – ≤ 23.5 versus those not at‐risk of malnutrition: 28.5% versus 7%, *p* < 0.05 [21].

#### Malnutrition

Malnutrition, defined by a BMI < 18.5 kg/m^2^, did not impact any reported outcome following primary THA and TKA, excluding a clinically insignificant 6‐min increase in operative time and a 0.3 day increase in LOHS within one primary THA study [3, 77].

A diagnosis of malnutrition via the short‐form MNA (SF‐MNA < 7) or MNA (score ≤ 11 in this instance) was associated with increased complication rate in both primary and revision cohorts, but statistical significance was only reached for those with a MNA ≤ 11 [29]. When analysing all primary and revision arthroplasty procedures performed at the study centre, both the SF‐MNA and MNA predicted increased LOHS: *p* = 0.001 for each [29].

When adjusted for preoperative scores, a SF‐MNA score < 7 predicted smaller improvements in role‐emotional and mental health components of the SF‐36 [29]. Adjusted scores were not reported for the MNA but a score ≤ 11 was associated with lower physical functioning, role physical, general health, vitality, role emotional and mental health sub‐scales [29].

Following arthroplasty for hip fracture, malnutrition defined as a Malnutrition Universal Screening Tool (MUST) score of ≤2 or MNA < 7 increased mortality at 1‐, 2‐, 6‐ and 12‐months [55]. When defined by a MNA < 17, malnutrition increased complication rate (25% vs. 7%, *p* < 0.05), accounting for 70% (62%–78%) of the differences seen compared to those without malnutrition [21].

#### Frailty

Frailty as per the modified frailty index (MFI) and moderate‐severe frailty as per the electronic frailty index (eFI) increased 30‐day mortality following primary THA and TKA [11, 59, 72]. One‐year mortality following primary TKA was also higher in the frail and vulnerable categories defined by the Frailty Index (FI) (4.08% and 0.96%), although statistical analyses were not performed relative to those without frailty (0%) [33]. Both the Groningen frailty index (GFI) and Veiligheids Management System Frailty Score (VMS) predicted overall mortality following arthroplasty for hip fracture, only the VMS predicted 30‐day mortality [73].

When defined as MFI ≤ 2, increased complication and SSI rates were seen among frail patients undergoing primary THA or TKA in association with longer LOHS [59, 72]. Frailty as per the eFI increased complication rates following primary THA (mild, moderate and severe) and primary TKA (moderate‐severe only) [12]. Following primary TKA, frailty as per the FI, Hospital Frailty Risk Score (HFRS) [≤ 5] and Comprehensive Geriatric Assessment‐Frailty Index (CGA‐FI) showed detrimental effect on complication rates, including increased rates of PJI when diagnosed using FI and HFRS [30, 33, 38].

Increased reoperation rates were associated with GFI scores ≤ 4 in primary THA but frailty as per the GFI, MFI and HFRS had no effect following primary TKA [38, 43, 59]. Frailty as per the FI had no significant effect on revision rates following primary TKA [33].

Despite lower baseline Hip Osteoarthritis Outcomes Score (HOOS) and Knee Osteoarthritis Outcomes Score (KOOS) scores among frail patients (GFI ≤ 4), the pain, symptoms and ADL sub‐scale scores showed equal improvement following primary THA and TKA; improvements in the sports sub‐scale scores were significantly higher among non‐frail patients [43]. Mild, moderate and severe frailty as per the eFI were associated with lower Oxford hip and knee scores when adjusted for age, sex, deprivation and pre‐op score following primary THA and TKA respectively [12]. Frailty defined by a VMS ≤ 2, but not via the GFI, was associated with increased ADL dependency/functional decline following arthroplasty for hip fracture [73].

Frailty, indicated by a HFRS score of 5 ≤ , increased LOHS (4.1 vs. 2.2 days, IRR 1.16 [1.16–1.17] *p* = 0.001) and cost ($19,841 vs. $6104) upon analyses of 882,479 primary TKAs within the NSQIP database [38].

In revision hip and knee cohorts, a HFRS score of ≤ 5 was associated with increased complication, re‐operation and PJI/SSI rate [37, 67].

#### Sarcopenia

Low Psoas:Lumbar Vertebrae Index (PLVI) increased rates of PJI in a combined cohort of primary arthroplasty patients and was more common among patients who died following arthroplasty for hip fracture: 58.4% versus 40%, *p* = 0.001 [1, 2].

Calf circumference and the SARC‐CalF assessment, which combines calf circumference with a questionnaire assessment, where both linked to reduced ability to walk 50 m independently following primary THA but not following primary TKA [46]. Sarcopenia, as per the appendicular skeletal muscle index, increased rates of blood transfusion in two primary TKA studies: 28.6% versus 4.7% and 5.7%, *p* < 0.001 for the former and OR 6.6 [1.57–45.4], *p* = 0.021 for the latter [27, 62].

Poorer functional outcomes and slower recovery were observed among sarcopenia patients following primary arthroplasty. Following primary THA, fewer patients with a low psoas muscle index achieved the minimal clinically important difference (MCID) for EQ5D, HOOS and VAS lower‐back pain scores [52]. After primary TKA, low skeletal muscle index was associated with lower KOOS ADL, Sports/Rec and QoL sub‐scales, increased pain, stiffness and functional limitation as per the WOMAC at 3‐ and 6‐months, and a lower chance of achieving the MCID for KOOS and PROMIS‐PF‐SF10 [25, 62].

#### Micronutrient deficiency

Zinc deficiency increased rates of delayed wound healing among those undergoing arthroplasty for hip fracture with delayed wound healing leading to longer LOHS [76].

### Non‐ESPEN criteria

Thirty‐six studies reported the effect of nutritional disorder following hip and/or knee arthroplasty but did not use ESPEN approved criteria [3, 4, 5, 6, 10, 13, 16, 17, 18, 19, 22, 24, 26, 31, 32, 34, 35, 40, 41, 42, 44, 47, 48, 49, 51, 55, 59, 61, 64, 65, 66, 68, 70, 72, 74, 75]. In all these studies, hypoalbuminaemia was in isolation or in combination with other serum markers to diagnose malnutrition (Supporting Information: File 3).

#### Mortality

Malnutrition definitions involving hypoalbuminaemia increased mortality in all primary THA [44, 70, 72], primary TKA [18, 31, 44, 47, 59], revision knee arthroplasty [32] and arthroplasty following hip fracture studies [6, 34, 35, 40, 55] but not in all‐form primary hip arthroplasty (THA and Hemiarthroplasty) or UKA [26, 64]. No mortality data was available for revision hip arthroplasty.

#### Complication

Increased complication rates were seen following primary THA, primary TKA, all‐form primary hip, UKA, revision hip, revision knee and arthroplasty following hip fracture but the findings from primary TKA were mixed with 2/4 studies finding no increase in complication rate among hypoalbuminaemic patients [4, 6, 10, 13, 16, 17, 18, 22, 24, 26, 29, 31, 32, 44, 47, 48, 49, 51, 59, 64, 65, 70, 72, 74].

#### Reoperation and revision

Increased reoperation rates were seen following primary THA [19], in combined cohorts of primary and revision hip and knee arthroplasty [13, 51] and following arthroplasty for hip fracture [49]. No differences were seen within the UKA cohort or the primary TKA studies when accounting for BMI [18, 26, 59]. Hypoalbuminaemia increased risk of septic revision following primary and revision arthroplasty [5, 41, 68, 75].

#### PJI/SSI

Hypoalbuminaemia predicted PJI and/or SSI in primary THA [48, 70, 72], primary TKA [18, 42, 47, 59], combined THA and TKA [3, 61], UKA [26] and revision hip and knee populations [5, 22, 32, 51]. No effect was seen in hip fracture patients [6, 49].

#### LOHS

Longer stays in hospital were found in hypoalbuminaemic patients undergoing primary THA and TKA [16, 19, 44, 70, 72], all‐form primary hip arthroplasty [64], UKA [26] and revision arthroplasty [32, 51]. Two of the three studies found hypoalbuminaemia increased LOHS in primary TKA patients [18, 44, 59]. In combined primary hip and knee populations, one study reported a significant increase in LOHS but in overweight and obese patients, hypoalbuminaemia had no detrimental effect [4, 13]. Two NSQIP studies found hypoalbuminaemia to increased LOHS following arthroplasty for hip fracture but a small cohort study from Turkey found no association [6, 35, 49].

#### Economic analyses

Hypoalbuminaemia, the chosen marker of malnutrition, increased resource utilisation in primary THA and TKA arthroplasty in those with BMI greater than 35 [16].

#### Co‐morbidity

Co‐morbid frailty and malnutrition (defined as MFI ≤ 2 and hypoalbuminaemia) had additive effect with the effect size on mortality, complication rate, reoperation and LOHS being greater within primary THA and primary TKA than for frailty or malnutrition alone [59, 72].

## DISCUSSION

This systematic review identified 58 studies that defined nutritional disorder and assessed the effects on chosen outcomes following hip and knee arthroplasty, 26 of which used criteria aligned with ESPEN recommendations [1, 2, 3, 11, 12, 21, 25, 27, 28, 29, 30, 33, 37, 38, 43, 46, 52, 55, 59, 60, 62, 67, 72, 73, 76, 77]. Through use of ESPEN criteria, a larger patient population and stratification of results by arthroplasty population and nutritional disorder, this article provides a more comprehensive summary of the literature than previous systematic review [7].

The MNA, SF‐MNA, MUST and NRS 2002 are ESPEN‐approved methods of diagnosing malnutrition or at‐risk of malnutrition, all four were associated with detrimental effect following primary, revision and hip fracture arthroplasty [8, 9, 21, 28, 29, 55, 60]. A BMI < 18.5 kg/m^2^ is a further ESPEN‐approved criteria for a diagnosis of malnutrition. Whilst this measure appears to be a poor predictor of adverse outcome following primary hip arthroplasty, the effects in higher risk populations such as hip fracture and revision arthroplasty populations are unknown [77]. Within the same arthroplasty population, different definitions of malnutrition had variable effect and whilst no power or sensitivity calculations were performed, future prospective study should take this into consideration [29].

In contrast, all definitions of frailty had detrimental effect following primary, revision and hip fracture arthroplasty, albeit no included study reported effect in UKA. A diagnosis of frailty therefore appears to identify individuals at higher risk of poorer outcomes, but the effect size and associated cost is still unclear given the heterogeneity in chosen definitions and limited economic analyses.

Sarcopenia, like frailty, is a nutritional disorder lacking a universal definition [9]. This systematic review identified four studies that used CT imaging to diagnose sarcopenia, each with a different diagnostic criterion but all showing detrimental effect in primary arthroplasty populations [25, 27, 52, 62]. Given concerns over radiation exposure, cost and availability of CT scanners, the effectiveness of non‐ionising techniques such as bioelectrical impedance analysis, anthropomorphic measurements and questionnaire assessment to diagnose sarcopenia and predict outcome following hip and knee arthroplasty must be considered [8, 9]. Only one included study used non‐ionising techniques to diagnose sarcopenia and no included study assessed the impact of sarcopenia in hip fracture or revision populations where there may be a more prominent effect [39, 46]. There is, however, growing interest into the impact of frailty and sarcopenia on lower limb arthroplasty populations given the number of articles which were published between September 2022 and March 2024 [12, 25, 27, 30, 33, 36, 37, 38, 46, 52, 62, 67].

Micronutrient deficiency is a category of nutritional disorder that can be assessed and addressed in a relatively simple and inexpensive manner but only one form of micronutrient deficiency was assessed by included studies [9, 76]. In this case, Zinc deficiency was associated with delayed wound healing which, in turn, was associated with longer LOHS, highlighting the potential benefit of addressing micronutrient deficiency within arthroplasty populations [76]. It remains unclear what other micronutrient deficits have detrimental effect in these patient populations.

Hypoalbuminaemia was the most common definition of nutritional disorder in this study (Table 3), consistent with prior systematic review [7, 39]. This definition has many limitations and ESPEN no longer recommends use of visceral proteins such as albumin as biomarkers for malnutrition [8, 9]. Patient factors that lead to hypoalbuminaemia will however increase risk of nutritional disorder [5, 9, 13, 21, 29, 41, 50, 57, 64, 75]. In this study, hypoalbuminaemia had effect on all outcome measures across all arthroplasty populations whilst demonstrating additive effect with frailty [59, 72]. We therefore suggest that whilst albumin is a poor marker of nutritional disorder, the prevalence and effect of hypoalbuminaemia in this systematic review re‐affirms the presence and detrimental effect of nutritional disorder within arthroplasty populations. Given previous reports, we did not think further pooled analysis on effects of hypoalbuminaemia would add to the literature [7].

**Table 3:** Definitions of nutritional disorder used by included studies.

**Questionnaire**
Mini Nutritional Assessment *N* = 2
Short‐form Mini Nutritional Assessment <7 *N* = 2
Short Nutritional Assessment Questionnaire 1 ≤ *N* = 1
Nutrition Risk Screening 2002 3 ≤ *N* = 4
Malnutrition Universal Screening Tool 2 ≤ *N* = 1
Geriatric Nutritional Risk Index: normal (98 < ), moderate malnutrition (92–98) and severe malnutrition ( < 92) *N* = 1
Graz Malnutrition Screening 3 ≤ *N* = 1
Modified Frailty Index (5‐item) 2 ≤ *N* = 2
Groningen Frailty Indicator 4 ≤ *N* = 2
Electronic Frailty Index: Fit (≤0.12), Mild Frailty (0.12 < ‐ ≤ 0.24) Moderate Frailty (0.24 < ‐ ≤ 0.36) and Severe Frailty (>0.36) *N* = 2
Veiligheids Management System Frailty Score 2 ≤ *N* = 1
Comprehensive Geriatric Assessment‐Frailty Index (CGA‐FI) 0.25 < *N* = 1
Frailty Index: non‐frail (<0.11), vulnerable (0.11–0.2) and frail (0.2<) *N* = 1
Hospital Frailty Risk Score 5 ≤ *N* = 3
**Anthropomorphic measurement**
Body Mass Index <18.5 kg/m^2^ *N* = 5
Psoas‐Lumbar Vertebrae Index *N* = 1
Skeletal Muscle Index <39 cm^2^/m^2^ (Male) or <53 cm^2^/m^2^ (Female)
Appendicular Skeletal Muscle Index <7 kg/m^2^ (Male) or <5.7 kg/m^2^ (Female) *N* = 1 and <7 kg/m^2^ (Male) or <5.4 kg/m^2^ (Female) *N* = 1
Psoas Muscle Index <6.36 cm^2^/m^2^ (Male) and <3.92 cm^2^/m^2^ (Female) *N* = 1
Calf circumference <34 cm (Male) and <33 cm (Female) *N* = 1
**Serum biomarkers**
Hypoalbuminaemia (<35 g/L) *N* = 32
Total Lymphocyte Count <1.5 ×109/L *N* = 5
Platelet Count <150×10^4^/mL *N* = 2
Haemoglobin <120 g/L *N* = 2
White Cell Count <4×10^9^/L *N* = 2
Prognostic Nutritional Index <48.4 *N* = 2 and <43.85 *N* = 1
Serum Transferrin <200 mg/dL *N* = 2
Albumin <37 g/L *N* = 1
Total Lymphocyte Count <1.2 ×10^9^/L *N* = 1
Protein Energy Malnutrition (Hypoalbuminaemia <3.5 g/L and Total Lymphocyte Count <1.5 ×109/L) *N* = 2
Stratified Serum Albumin *N *= 1
Platelet:White Cell Count ≤28.57 *N* = 1
Serum Albumin:Fibrinogen (1/4 Albumin:Fibrinogen g/L) *N* = 2
Serum Zinc <95 μg/dL *N* = 1
**Combination**
SARC‐CalF: calf circumference + SARC‐F screening questionnaire *N* = 1

In addition to the infrequent use of ESPEN diagnostic criteria, the lack of economic analyses is a key limitation of published literature and, by extension, this systematic review. Only one study reported the effect of an ESPEN‐recommended definition of nutritional disorder (frailty) on healthcare costs [38]. This study found frail patients incurred a greater than threefold increase in costs following primary TKA, likely secondary to the increased LOHS and rates of complication, re‐admission, PJI and re‐operation [38]. Whilst this systematic review cannot quantify costs for each nutritional disorder and arthroplasty population, the association seen between nutritional disorder and LOHS, complication rate, PJI and re‐operation, suggests increased healthcare costs are incurred when performing arthroplasty on these patients, particularly revision and hip fracture cases. Further limitations of this systematic review include limited PROMs data, a lack of studies using ESPEN criteria in UKA and obese populations, and limited reporting of revision rates, a core outcome associated with poorer patient outcomes and increased cost [14, 29, 43, 54, 56, 58, 69, 73]. In combination with heterogeneity in study demographics and biases, these limitations prevented pooled analyses and calculation of hazard or risk ratios as outlined in the study proposal. This study was therefore limited in its ability to determine the full effect of each nutritional disorder across different arthroplasty populations. In relation, our protocol excluded studies with <50 cases given well established biases associated with small samples sizes and planned meta‐analyses. By excluding these studies, some effects of nutritional disorder may not have been reported, although with over 2 million included patients, the findings of this systematic review are still robust within the limitations of the published literature.

## CONCLUSION

We conclude that nutritional disorder has detrimental effects on clinical outcomes following hip and knee arthroplasty. Whilst economic analyses were limited, the observed effect of nutritional disorders on adverse outcomes makes it likely that increased healthcare costs are associated with hip and knee arthroplasty in these populations. Further prospective work is required to understand the potential for cost‐saving and improvements in patient outcomes that may come from the treatment of nutritional disorder within each hip and knee arthroplasty population.

## AUTHOR CONTRIBUTIONS

Christopher David John Little co‐designed the review, assisted in the formulation of the search strategy, conducted the search, evaluated articles for inclusion, extracted the data, performed the methodological quality assessment, and wrote the manuscript. Hariharan Subbiah Ponniah extracted the data, evaluated articles for inclusion, performed the methodological assessment and assisted in manuscript preparation. Thomas William Hamilton co‐designed the review and assisted in the formation of the search strategy, assessment of articles for inclusion and preparation of the manuscript for submission. Louise Strickland assisted in the formation of the search strategy and edited the manuscript prior to submission. Shvaita Ralhan and Antony Palmer reviewed and edited the manuscript prior to submission.

## CONFLICT OF INTEREST STATEMENT

The authors declare no conflict of interest.

## ETHICS STATEMENT

PROSPERO ID: CRD42023360517. There is no ethical approval needed for this systematic review. There is no informed consent needed for this systematic review.

## DECLARATION

Figure 1 is the property of one of the authors. The graphical abstract was generated by one of the authors using an open AI source (Chat GPT) and is original in nature.

## Supporting information


**Additional File 1.** Format: Word Document. Title: Search Strategy. Description of data: Search strategies for each database.

**Additional File 2.** Format: Word Document. Title: Study Demographics. Description of Data: Table containing the study demographics for each included study.

**Additional File 3.** Format: Word Document. Title: Non‐ESPEN Outcomes. Description of Data: Table containing outcomes for definitions of nutritional disorder that do not satisfy ESPEN criteria.

## Data Availability

All data used in this systematic review is available via the referenced articles.
