# Supplementary material for: Nutritional disorders have adverse effect on clinical outcomes and may increase healthcare costs following hip and knee arthroplasty—A systematic review
Source: J Exp Orthop. 2026 Oct 15;13(4):e70657. doi: 10.1002/jeo2.70657 (PMC13671359; doi:10.1002/jeo2.70657)
Supplement: Supplementary file 1 [file JEO2-13-e70657-s001.docx]

| **Population** | **Outcome** | **Definition Nutritional Disorder** | **N=** | **Nutritional disorder** | **Comparator** | **Effect size(s) Nutritional Disorder** | **Other data reported** | **References** |
| --- | --- | --- | --- | --- | --- | --- | --- | --- |
| Primary Total Hip Arthroplasty | Mortality - 30-day | Hypoalbuminaemia | **39,312** | **Walls et al 2015**  1.25% | **Walls et al 2015**  0.13% | **Walls et al 2015** OR 5.94 [3.07-11.48]  **Meyer et al 2017** OR 7.81 [1.41-43.23] | / | (Walls et al., 2015, Meyer et al., 2017) |
|  |  | Hypoalbuminaemia and Modified Frailty Index 2≤:  Malnourished  Malnourished + Frail | **105,997** | 0.7%  1.9% | 0.10% | OR 4.93 [3.08-7.88]  OR 12.66 [8.81-20.83]  p<0.001 for both | / | (Wilson et al., 2020) |
|  | Complication Rate | Hypoalbuminaemia | **59,522** | / | / | **Fu *et al.* 2016**  Overall: OR 1.61 [1.25-2.08] p<0.001  Wound complications: OR 2.35 [1.54 -3.59] p<0.001  **Meyer *et al.* 2017**  Wound complications: OR 3.58 [1.83-6.99] p<0.001  **Walls *et al.* 2015**  Pneumonia OR 2.41 [1.34–4.32]  Urinary tract infection OR 1.63 [1.11–2.39]  Bleeding transfusion OR 1.82 [1.59–2.09]  Any infection OR 2.02 [1.58–2.58]  Cardiac pulmonary complications OR 1.66 [1.01–2.72]  Any major complications OR 1.89 [1.29–2.76] | / | (Fu et al., 2016, Meyer et al., 2017, Walls et al., 2015) |
|  |  | Hypoalbuminaemia and Modified Frailty Index 2≤:  Malnourished  Malnourished + Frail | **105,997** | Minor Complication  3.7%  7.1%  Major Complication  4.2%  7.3% | 1.6% for both | Minor Complication  2.01 [1.66-2.43] p<0.001  3.5 [2.75-4.44] p<0.001  Major Complication  2.41 [2.01-2.88] p<0.001  3.77 [2.98-4.81] p<0.001 | / | (Wilson et al., 2020) |
|  |  | Protein Energy Malnutrition: Hypoalbuminaemia <35g/L + Total Lymphocyte Count <1.5 x 10^9^/L | **220** | 44.4% | 7.8% | HR 6.3 [1.7-23.1] | / | (Eminovic et al.) |
|  |  | Hypoalbuminaemia:  <2.75 g/dL  ⩾2.75 – <3.0 g/dL  ⩾3.0 – <3.25 g/dL: | **24,586** | / | / | Major Complication  OR 1.97 [1.34-2.92] p=0.001  OR 1.86 [1.18-2.95] p=0.008  OR 1.37 [1.02-1.85] p=0.039 | / | (Nelson et al., 2019) |
|  |  | Prognostic Nutritional Index <43.85: Albumin (g/L) + 5x total lymphocyte counts (10^9^/L) | **254** | / | / | Post-op Delirium  OR 3.47 [1.2-10.04] p=0.022 | / | (Hu et al., 2023) |
|  | Re-operation Rate | Hypoalbuminaemia | **20,210** | / | / | <30 days  OR 1.70 [1.15-2.53] p=0.008 | / | (Fu et al., 2016) |
|  |  | Hypoalbuminaemia and Modified Frailty Index 2≤:  Malnourished + Frail  Malnourished | **105,997** | 5.5%  3.7% | 1.9% | OR 2.53 [1.92-3.34] p<0.001  OR 1.84 [1.52-2.23] p<0.001 |  | (Wilson et al., 2020) |
|  | No data available for revision rate | | | | | | | |
|  | PJI/SSI Rate | Protein Energy Malnutrition: Hypoalbuminaemia <35g/L + Total Lymphocyte Count <1.5 x 10^9^/L | **220** | PJI = 0% | PJI = 0.5% | / | / | (Eminovic et al.) |
|  |  | Hypoalbuminaemia:  <2.75 g/dL  ⩾2.75 – <3.0 g/dL  ⩾3.0 – <3.25 g/dL: | **24,586** | / | / | Adjusted OR for wound infection:  2.19 [0.90-5.35] p=0.086  2.44 [0.91-6.55] p=0.077  1.07 [0.46-2.51] p=0.871 | / | (Nelson et al., 2019) |
|  |  | Hypoalbuminaemia | **24,238** | Surgical SSI: 2.14%  Deep incisional SSI: 0.71%  Organ Space SSI: 0.27% | Surgical SSI: 0.71%  Deep incisional SSI: 0.27%  Organ Space SSI: 0.2% | Surgical SSI: OR 2.61 [1.68–4.06]  Deep Incisional SSI: OR 2.35 [1.12–4.91] | / | (Walls et al., 2015) |
|  | No PROM data available | | | | | | | |
|  | LOHS (days) | Hypoalbuminaemia | **59,522** | **Walls *et al.* 2015**  4.67 ± 6.05 | **Walls *et al.* 2015**  3.18 ± 4.13 | **Walls *et al.* 2015** P<0.01  **Fu *et al.* 2016**  Extended LOHS: OR 1.35 [1.14 -1.59]; p=0.001  **Meyer *et al.* 2017**  Prolonged LOHS (>75^th^ %) OR 3.50 [1.79–6.85] p<0.001 | / | (Fu et al., 2016, Meyer et al., 2017, Walls et al., 2015) |
|  |  | Hypoalbuminaemia and Modified Frailty Index 2≤:  Malnourished + Frail  Malnourished | **105,997** | Extended >2 days  73.3%  57.8% | Extended >2 days  40.4% | OR 3.14 [2.73-3.63] p<0.001  OR 1.71 [1.59-1.84] p<0.001 | / | (Wilson et al., 2020) |
|  |  | Protein Energy Malnutrition: Hypoalbuminaemia <35g/L + Total Lymphocyte Count <1.5 x 10^9^/L | **220** | Preoperative  1 (1-36) days  Postoperative  14 (4-36) days | Preoperative  1 (1-22) days  Postoperative  14 (1-50) days | P<0.001 for preoperative  P<0.544 for postoperative | / | (Eminovic et al.) |
|  | No data available for economic analysis | | | | | | | |
| Primary Total knee Arthroplasty | Mortality | Hypoalbuminaemia | **140,622** | **Johnson *et al.* 2021** 1%  **Nelson *et al.* 2015** 0.64%  **Fu *et al.* 2017** 0.6% | **Johnson *et al.* 2021** 0.1%  **Nelson *et al.* 2015** 0.15%  **Fu *et al.* 2017** 0.1% | **Meyer *et al.* 2017** OR 9.23 [2.51-34.09]  **Johnson *et al.* 2021** OR 7.2 [4.5-11.55]  **Nelson *et al.* 2015** OR [1.58-6.35] p=0.001  **Fu *et al.* 2017** OR 3.17 [1.46-6.9] p=0.004 | / | (Johnson et al., 2021, Meyer et al., 2017, Fu et al., 2017, Nelson et al., 2015) |
|  |  | Hypoalbuminaemia and Modified Frailty Index 2≤:  Malnourished + Frail  Malnourished | **179,702** | 1%  0.3% | 0.1% | OR 9.43 [5.92-14.93] p<0.001  OR 2.97 [1.76-5.01] p<0.001 | / | (Schwartz et al., 2020) |
|  | Complication Rate | Hypoalbuminaemia and Modified Frailty Index 2≤:  Malnourished + Frail  Malnourished | **179,702** | 8.7%  5.2% | 3.4% | OR 2.40 [2.06-2.79] p<0.001  OR 1.44 [1.27-1.63] p<0.001 | / | (Schwartz et al., 2020) |
|  |  | Hypoalbuminaemia | **140,622** | **Johnson *et al.* 2021** 17%  **Nelson *et al.* 2015** Major 2.36%  **Fu *et al.* 2017**  Any 7.7%  Major 5.4% | **Johnson *et al.* 2021** 5%  **Nelson *et al.* 2015** 1.42%  **Fu *et al.* 2017**  Any 5%  Major 3.7% | **Meyer *et al.* 2017** OR 1.56 [95%CI 0.82-2.98]; p=0.175  **Johnson *et al.* 2021** OR 3.67 [3.28-4.2]  **Nelson *et al.* 2015** OR 1.41 [1-1.97] p=0.05  **Fu *et al.* 2017** Any 1.37 [1.11-1.68] p=0.003  Major 1.32 [1.04-1.68] p=0.024 | / | (Johnson et al., 2021, Meyer et al., 2017, Fu et al., 2017, Nelson et al., 2015) |
|  | Re-operation Rate | Hypoalbuminaemia and Modified Frailty Index 2≤:  Malnourished + Frail  Malnourished | **179,702** | 3.0%  1.9% | 1% | OR 2.66 [2.06-3.44] p<0.001  OR 1.75 [1.42-2.15] p<0.001 | / | (Schwartz et al., 2020) |
|  |  | Hypoalbuminaemia | **34,800** | 1.6% | 1.2% | OR 1.1 [0.71-1.71] p=0.658 | / | (Fu et al., 2017) |
|  | No data available for revision rate | | | | | | | |
|  | PJI/SSI Rate | Hypoalbuminaemia | **37,767** | **Nelson et al. 2015**  Superficial SSI 1.27%  Deep SSI 0.38%  Organ Space SSI 0.45%  **Man et al. 2020**  PJI 11.8% | **Nelson et al. 2015**  Superficial SSI 0.64%  Deep SSI 0.12%  Organ Space SSI 0.15%  **Man et al. 2020**  PJI 0.5% | **Nelson et al. 2015**  Superficial SSI OR 1.27 [1.09–2.75] p=0.020  Deep SSI OR 3.64 [1.54–8.63] p=0.003  Organ Space SSI OR 2.71 [1.23–5.97] p=0.013 | / | (Nelson et al., 2015, Man et al., 2020) |
|  |  | Hypoalbuminaemia and Modified Frailty Index 2≤:  Malnourished + Frail  Malnourished | **179,702** | Superficial SSI  1.1%  0.7%  Deep SSI  0.66%  0.2% | Superficial SSI 0.5%  Deep SSI 0.2% | Superficial SSI  OR 1.93 [1.28-2.92] p=0.02  OR 1.44 [1.04-1.99] p=0.030=  Deep SSI  OR 3.27 [1.96-5.43] p<0.001  OR 1.92 [1.23-3.01] p=0.004 | / | (Schwartz et al., 2020) |
|  | No PROM data available | | | | | | | |
|  | LOHS | Hypoalbuminaemia and Modified Frailty Index 2≤:  Malnourished + Frail  Malnourished | **179,702** | >2 days  66.7%  57.6% | >2 days  48.4% | OR 1.91 [1.75-2.09] p<0.001  OR 1.31 [1.24-1.39] p<0.001 |  | (Schwartz et al., 2020) |
|  |  | Hypoalbuminaemia | **58,656** | **Fu *et al.* 2017** >4 days  14.2% | **Fu *et al.* 2017** >4 days  8.6% | **Fu *et al.* 2017** P<0.001  **Meyer *et al.* 2017** >75^th^ percentile  OR 1.80 (0.87–3.72) p=0.112 | / | (Fu et al., 2017, Meyer et al., 2017) |
|  | No data available for economic analysis | | | | | | | |
| All-form primary Hip Arthroplasty (Total and Hemiarthroplasty) | Mortality | Hypoalbuminaemia | **211** | 1.3% | 0% | OR 0 [0-5.5] p=0.2 | / | (Tan et al., 2021) |
|  | Complication Rate | Hypoalbuminaemia | **211** | 62.5% | 45% | OR 0.49 [0.28-0.87] p=0.014 | Delayed Wound Healing  25% vs 10.7%  OR 0.36 [0.17-0.76] p=0.006 | (Tan et al., 2021) |
|  | No data available for re-operation, revision or PJI/SSI Rate and no PROM data reported | | | | | | | |
|  | LOHS (days) | Hypoalbuminaemia | **211** | 14.99 ± 5.72 | 12.46 ± 3.16 | P<0.001 | / | (Tan et al., 2021) |
|  | No data available for economic analysis | | | | | | | |
| Unicompartmental Knee Arthroplasty | Mortality | Hypoalbuminaemia | **6049** | 0% | 0.05% | Not reported | / | (Hur et al., 2020) |
|  | Complication Rate | Hypoalbuminaemia | **6049** | 7.02% | 2.23% | Adjusted RR 2.2 [1.2-4] p=0.009 | / | (Hur et al., 2020) |
|  | Re-operation Rate | Hypoalbuminaemia | **6049** | 2.34% | 0.91% | Adjusted RR 2.0 [0.8–5.2] p= 0.148 | / | (Hur et al., 2020) |
|  | No data available for revision rate | | | | | | | |
|  | PJI/SSI Rate | Hypoalbuminaemia | **6049** | SSI 2.34 | SSI 0.75 | RR 2.7 [0.9-7.5] p=0.063 | / | (Hur et al., 2020) |
|  | No PROM data available | | | | | | | |
|  | LOHS (days) | Hypoalbuminaemia | **6049** | 2.2 | 1.8 | p=0.031 | / | (Hur et al., 2020) |
|  | No data available for economic analysis | | | | | | | |
| Primary Total Hip and Knee Arthroplasty (combined cohorts) | No data available for mortality | | | | | | | |
|  | Complication Rate | Hypoalbuminaemia | **130,057** | **Bohl *et al.* 2016b**  7.3%  **Courtney *et al.* 2016**  BMI >40 29%  BMI <40 25% | **Bohl *et al.* 2016b**  4%  **Courtney *et al.* 2016**  BMI >40 7%  BMI <40 8% | **Bohl *et al.* 2016b**  Adjusted RR 1.5 [1.2-1.7] p<0.001  **Courtney *et al.* 2016**  Hypoalbuminaemia OR 3 [1.56-5.75] p<0.001  **Holbert *et al.* 2023**  BMI 35 - <40: OR 1.51 [1.23-1.86] p<0.001  BMI 40 - <45: OR 1.6 [1.23-2.04] p<0.001  BMI 45 - <50: OR 1.54 [1.03-2.22] p=0.027  BMI 50≤: OR 1.71 [1.05-2.69] p=0.026 | **Bohl *et al.* 2016b**  Hypoalbuminaemia associated with higher rate of serious complication: 2.1% vs 1.2%, adjusted RR 1.4 [1.3-2.5] p<0.001  **Courtney *et al.* 2016**  Malnourished more likely to require ICU intervention (14 vs 2%; p<0.001) and readmission within 90 days (17 vs 4%; p<0.001) | (Bohl et al., 2016b, Courtney et al., 2016, Holbert et al., 2023) |
|  |  | Prognostic Nutritional Index (PNI): 10 × serum albumin (g/dL) + 0.005 × total lymphocyte count (/µL) | **994** | / | / | Postoperative Delirium  OR 0.908 [95% CI 0.840-0.983] p=0.017 | Sensitivity: 59.7% Specificity: 71.4% Positive predictive value: 13.1% Negative predictive value: 96.1% | (Chen et al., 2021) |
|  | Re-operation Rate | Hypoalbuminaemia | **670** | 8% | 3% | p=0.008 | / | (Courtney et al., 2016) |
|  | No data available for revision rate | | | | | | | |
|  | PJI/SSI Rate | Hypoalbuminaemia | **81,136** | **Blevins *et al.* 2018**  PJI 11.6%  **Bohl *et al.* 2016b**  SSI 2.29% | **Blevins *et al.* 2018**  PJI 1.9%  **Bohl *et al.* 2016b**  SSI 0.96% | **Blevins *et al.* 2018**  Adjusted OR 4.697 [2.428-9.085] p<0.001  **Bohl *et al.* 2016b**  Adjusted RR 2 [1.5-2.80 p<0.001 | **Courtney *et al.* 2016**  Hypoalbuminaemic patients more like to return to theatre for infection: 6% vs 0.3%, p=0.004 | (Blevins et al., 2018, Bohl et al., 2016b, Courtney et al., 2016) |
|  |  | White Cell Count <4x109/L | **30,863** | 2.2% | 1.9% | p=0.673 | / | (Blevins et al., 2018) |
|  |  | Platelet Count <150x104/mL | **30,863** | 6.7% | 3.5% | Adjusted OR 1.472 [0.729-2.972] p=0.281 | / | (Blevins et al., 2018) |
|  |  | Haemoglobin <120 g/L | **30,863** | 26.1% | 13.7% | Adjusted OR 1.729 [1.1-2.718] p=0.018 | / | (Blevins et al., 2018) |
|  |  | Platelet:White Cell Count ≤28.57 | **30,863** | 34.7% | 24.8% | Adjusted OR 1.227 [0.799-1.885] p=0.351 | / | (Blevins et al., 2018) |
|  |  | Albumin:Fibrinogen (AFR) <12.96 | **1791** | Wound Infection 5.8%  Superficial SSI 3.7%  Deep SSI 1.1%  PJI 1.4% | Wound Infection 2%  Superficial SSI 0.8%  Deep SSI 0.2%  PJI 0.4% | HR adjusted for age, BMI, surgery, operation time, Hb, ASA score, diagnosis and CCI:  Wound Infection  HR 3.772 [2.157-6.597] p<0.001  Superficial SSI  HR 4.097 [2.113-7.945] p<0.001  Deep SSI  HR 8.451 [1.612-44.297] p=0.012  PJI  HR 4.029 [1.523-16.6] p=0.008 | / | (Shang et al., 2023) |
|  | No PROM data available | | | | | | | |
|  | LOHS (days) | Hypoalbuminaemia | **130,057** | **Holbert *et al.* 2023**  2.94 ± 3.93  3≤ days  18.9% | **Holbert *et al.* 2023**  2.12 ± 1.98  3≤ days  9.4% | **Holbert *et al.* 2023**  P<0.001 for both | **Bohl *et al.* 2016b**  Postoperative Length of stay longer among hypoalbuminaemic patients: 3.52 vs 3.10 days; adjusted difference 0.20 days [0.12-0.27] p<0.001.  **Courtney *et al.* 2016**  Length of Stay BMI >40 vs <40  Nourished: 3.29 days vs 3.29 days; p=0.966  Malnourished 3.67 days vs 3.98 days p=0.616 | (Bohl et al., 2016b, Courtney et al., 2016, Holbert et al., 2023) |
|  | Economic Analysis | Hypoalbuminaemia | **78,784** | / | / | Hypoalbuminaemia as a predictor of resource utilisation:  BMI 35 - <40: OR 1.82 [1.64-2] p<0.001  BMI 40 - <45: OR 1.39 [1.22-1.580 p<0.001  BMI 45 - <50: OR 1.28 [1.04-1.58] p=0.018  BMI 50≤: OR 1.73 [1.33-2.260 p<0.001 | **/** | (Holbert et al., 2023) |
| Revision Hip Arthroplasty | No data available for mortality | | | | | | | |
|  | Complication Rate | Hypoalbuminaemia | **12,249** | / | / | Urinary tract infection  OR 1.840 [1.339-2.528] p<.001  Blood transfusion  OR 2.484 [2.250-2.743] p<0.001  Sepsis  OR 3.514 [2.618-4.716] p<.001  Septic shock  OR 5.976 [3.344-10.681] p<0.001  Pneumonia  OR 1.702 [1.128-2.567] p=0.011  Unplanned reintubation  OR 1.907 [1.084-3.355] p=0.025  Ventilation >48 hours  OR 2.478 [1.199-5.121] p=0.014  DVT/thrombophlebitis  OR 2.598 [1.713-3.938] p<0.001  Pulmonary embolism  OR 2.048 [1.100-3.811 p=0.024  AKI  OR 3.061 [1.283-7.305] p=0.012 | / | (Heimroth et al., 2023) |
|  | Re-operation Rate | Hypoalbuminaemia  Malnutrition: Hypoalbuminaemia and Total Lymphocyte Count <1500/mm^3^ | **90** | **Septic Failure**  Hypoalbuminaemia 26.8%  Malnutrition 17.1% | **Septic Failure**  Hypoalbuminaemia 10.2%  Malnutrition 6.1% | Hypoalbuminaemia p=0.04  Malnutrition p=0.1 | / | (Traverso et al., 2023) |
|  | No data available for revision rate | | | | | | | |
|  | PJI/SSI Rate | Hypoalbuminaemia | **12,249** | / | / | SSI  OR 2.240 [1.812-2.769] p<0.001 | / | (Heimroth et al., 2023) |
|  | No data available for PROM, LOHS or economic analysis | | | | | | | |
| Revision Knee Arthroplasty | Mortality | Hypoalbuminaemia | **4551** | 1.83% | 0.13% | OR 9.81 [3.38-28.49] p<0.001 | / | (Kamath et al., 2017) |
|  | Complication Rate | Hypoalbuminaemia | **4551** | 59.97% | 45.37% | OR 2.74 [2.21-3.39] p<0.001 | / | (Kamath et al., 2017) |
|  | Re-operation Rate | Hypoalbuminaemia  Malnutrition: Hypoalbuminaemia and Total Lymphocyte Count <1500/mm^3^ | **75** | **Septic Failure**  Hypoalbuminaemia 17.1%  Malnutrition 2.9% | **Septic Failure**  Hypoalbuminaemia 0%  Malnutrition 0% | Hypoalbuminaemia p=0.006  Malnutrition p=0.28 | / | (Traverso et al., 2023) |
|  | No data available for revision rate | | | | | | | |
|  | PJI/SSI Rate | Hypoalbuminaemia | **4551** | Superficial SSI 0.84%  Deep SSI 1.69%  Organ Space SSI 4.07% | Superficial SSI 0.69%  Deep SSI 0.81%  Organ Space SSI 1.25% | Superficial SSI  OR 1.35 [0.54-3.36] p=0.519  Deep SSI  OR 2.3 [1.15-4.6] p=0.019  Organ Space SSI  OR 3.79 [2.31-6.21] p<0.001 |  | (Kamath et al., 2017) |
|  | No PROM data available | | | | | | | |
|  | LOHS (days) | Hypoalbuminaemia | **4551** | 7.1 ± 7.5 | 3.6 ± 3.0 | p<0.001 | / | (Kamath et al., 2017) |
|  | No data available for economic analysis | | | | | | | |
| Revision Hip and Knee Arthroplasty (combined cohorts) | No data available for mortality | | | | | | | |
|  | Complication rate | Geriatric Nutritional Risk Index = (1.489 x serum albumin) + (41.7 x [body weight/ideal weight])  Moderate Malnutrition 92–98  Severe Malnutrition < 92 | **341** | **Any complication <90-days**  Moderate 22.2%  Severe 37.5%  **Re-admission**  Moderate 18.5%  Severe 35% | **Any complication <90-days**  19.2%  **Re-admission**  15.1% | **Any complication <90-days**  P=0.023  **Re-admission**  P=0.005 | / | (Oakley et al., 2023) |
|  | Reoperation rate | Hypoalbuminaemia | **165** | / | / | **Multivariate analysis for septic failure requiring re-operation**  OR 5.64 [1.26–25.18] p=0.023 | / | (Traverso et al., 2023) |
|  |  | Geriatric Nutritional Risk Index = (1.489 x serum albumin) + (41.7 x [body weight/ideal weight])  Moderate Malnutrition 92–98  Severe Malnutrition < 92 | **341** | Moderate: 29.4%  Severe: 50.0% | 21.9% | p=0.156 | / | (Oakley et al., 2023) |
|  | Revision Rate | Hypoalbuminaemia | **4983** | / | / | / | Hypoalbuminaemic patients more likely to have a septic revision  **Bohl *et al.* 2016a**  42.8% vs 11.8% Adjusted RR 3.8 [3.4-4.3] p<0.001  **Maimaiti *et al.* 2021**  80.2% vs 50% Adjusted OR 1.56 [1.16-2.08] p=0.003 | (Bohl et al., 2016a, Maimaiti et al., 2021) |
|  |  | Hypoalbuminaemia (<35g/L) **OR** Total Lymphocyte Count <1.5 x 10^9^/L **OR** Serum Transferrin <200 mg/dl) | **501** | / | / | / | Malnutrition was observed in 53% of septic revisions and 33% of aseptic revisions  OR 2.3 [1.5-3.5] p=0.003 upon multivariate analysis | (Yi et al., 2015) |
|  |  | Total Lymphocyte Count (TLC) <1.5 x 10^9^/L  White Cell Count (WCC) <4x10^9^/L  Platelet Count <150x10^4^/mL  Haemoglobin (Hb) <120 g/L  Serum 1/4 Albumin:Fibrinogen (AFR) <11.7  Prognostic Nutritional Index (PNI) <48.4 | **466** | / | / | / | OR for septic revision on multivariate analyses:  AFR: 2.54 [1.93-3.36] p<0.001  PNI <48.4: 1.57 [1.01-2.43] p=0.043  TLC, WCC, platelets and Hb had no significant effect | (Maimaiti et al., 2021) |
|  |  | Geriatric Nutritional Risk Index = (1.489 x serum albumin) + (41.7 x [body weight/ideal weight])  Moderate Malnutrition 92–98  Severe Malnutrition < 92 | **341** | **1-year all-cause revision**  Moderate 23.5%  Severe 45.5%  **2-year all-cause revision**  Moderate 23.5%  Severe 45.5% | **1-year all-cause revision**  16.6%  **2-year all-cause revision**  20% | 1-year p=0.001  2-year p=0.002 | / | (Oakley et al., 2023) |
|  | PJI/SSI Rate | Hypoalbuminaemia | **4517** | 4.5% | 2.1% | Adjusted RR 2.1 [1.3-3.5] p=0.002 | / | (Bohl et al., 2016a) |
|  |  | Hypoalbuminaemia (<35g/L) **OR** Total Lymphocyte Count <1.5 x 10^9^/L **OR** Serum Transferrin <200 mg/dl) | **501** | / | / | / | Malnutrition was independently associated with acute postoperative infection in aseptic revision arthroplasty; OR 5.9 [1.317-26.057] p=0.02 | (Yi et al., 2015) |
|  |  | Geriatric Nutritional Risk Index = (1.489 x serum albumin) + (41.7 x [body weight/ideal weight])  Moderate Malnutrition 92–98  Severe Malnutrition < 92 | **341** | 1-year PJI  Moderate 17.6%  Severe 18.2% | 1-year PJI  9.3% | p=0.281 | / | (Oakley et al., 2023) |
|  | LOHS (days) | Geriatric Nutritional Risk Index = (1.489 x serum albumin) + (41.7 x [body weight/ideal weight])  Moderate Malnutrition 92–98  Severe Malnutrition < 92 | **341** | Moderate: 5.6 (range 1.0–13.9)  Severe: 8.4 (range: 1.3–36.3) | Normal 4.7 (range: 1.0–33.2) | P<0.001 | / | (Oakley et al., 2023) |
|  | No data available for PROM or economic analysis | | | | | | | |
| Hip Fracture | Mortality | Hypoalbuminaemia | **19,556** | **Bohl *et al.* 2017**  30-day 9.94%  **Kieffer et al. 2013**  1-year 32.9%  **Kurtoglu *et al.* 2021**  Overall 25.5%  **Popp *et al.* 2023**  1 month 6.55%  3 month 13.61%  6 month 18.61%  12 month 25.16% | **Bohl *et al.* 2017**  30-day 5.53%  **Kieffer et al. 2013**  1-year 22.4%  **Kurtoglu *et al.* 2021**  Overall 11%  **Popp *et al.* 2023**  1 month: 2.99%  3 month 4.98%  6 month 7.64%  12 month 13.95% | **Bohl *et al.* 2017**  Adjusted RR 1.52 [1.37-1.7] p<0.001  **Kieffer et al. 2013**  OR 1.7 [1.16-2.5] p=0.0049  **Kurtoglu *et al.* 2021**  p=0.018  **Popp *et al.* 2023**  p<0.01 | / | (Bohl et al., 2017, Kieffer et al., 2013, Kurtoglu et al., 2021, Popp et al., 2023) |
|  |  | Albumin levels below <35 g/L **AND** Total Lymphocyte Count <1,500/mL | **109** | 36.1% | 8.2% | OR 4.64 [1.744-12.362] | / | (Lu et al., 2021) |
|  | Complication Rate | Hypoalbuminaemia | **6093** | **Newman *et al.* 2020**  40.1% | **Newman *et al.* 2020**  27.4% | **Newman *et al.* 2020**  OR 1.8 [1.43-2.26] p<0.0001  **Tian *et al.* 2022 - Pneumonia**  OR 6.18 [3.15-11.98] p<0.001  **Yao *et al.* 2023 - UTI**  OR 1.86 [1.28–2.70] | / | (Newman et al., 2020, Tian et al., 2022, Yao et al., 2023) |
|  |  | Serum albumin <3.7g/dL  Peripheral lymphocyte count <1200/μL | **152** | / | / | Post-operative delirium  OR 0.17 [0.03-0.86] p=0.0329 | Sensitivity 75% Specificity 56%  AUC 0.72  Peripheral lymphocyte count had no effect on rates of post-op delirium | (Tanaka, 2016) |
|  | Re-operation Rate | Hypoalbuminaemia | **1907** | **Newman *et al.* 2020**  6.2%  **Kurtoglu *et al.* 2021**  13.7% | **Newman *et al.* 2020**  3.4%  **Kurtoglu *et al.* 2021**  6.7% | **Newman *et al.* 2020**  OR 1.97 [1.2-3.23] p=0.007  **Kurtoglu *et al.* 2021**  p=0.108 | / | (Newman et al., 2020, Kurtoglu et al., 2021) |
|  | No data available for revision rate | | | | | | | |
|  | PJI/SSI Rate | Hypoalbuminaemia | **19,318** | **Bohl *et al.* 2017**  SSI 1.4%  **Newman 2020**  Superficial SSI 0.7%  Deep SSI 0.7%  Organ Space infection 0.7% | **Bohl *et al.* 2017**  SSI 1.18%  **Newman 2020**  Superficial SSI 1%  Deep SSI 0.4%  Organ Space infection 0.1% | **Bohl *et al.* 2017**  SSI RR 1.19 [0.91-1.56] p=0.195  **Newman 2020**  Superficial SSI OR 0.65 [0.17-2.45] p=0.52  Deep SSI OR 1.91 [0.46-7.95] p=0.372  Organ Space infection OR 8.74 [0.96-79.95] p=0.05 |  | (Bohl et al., 2017, Newman et al., 2020) |
|  | No PROM data available | | | | | | | |
|  | LOHS (days) | Hypoalbuminaemia | **19,558** | **Bohl *et al.* 2017**  5.67 ± 4.68  **Newman *et al.* 2020**  7  **Kurtoglu *et al.* 2021**  7 (4-22) | **Bohl *et al.* 2017**  4.99 ± 3.95  **Newman *et al.* 2020**  5  **Kurtoglu *et al.* 2021**  7 (4-22) | **Bohl *et al.* 2017**  Adjusted difference 0.5 [0.38-0.63] p<0.001  **Newman *et al.* 2020**  P<0.0001  **Kurtoglu *et al.* 2021**  p=0.309 |  | (Bohl et al., 2017, Newman et al., 2020, Kurtoglu et al., 2021) |
|  | No data available for economic analysis | | | | | | | |
| Primary and Revision Hip and Knee Arthroplasty | No data available for any outcome of interest | | | | | | | |

BLEVINS, K., AALIREZAIE, A., SHOHAT, N. & PARVIZI, J. 2018. Malnutrition and the Development of Periprosthetic Joint Infection in Patients Undergoing Primary Elective Total Joint Arthroplasty. *Journal of Arthroplasty,* 33**,** 2971-2975.

BOHL, D. D., SHEN, M. R., HANNON, C. P., FILLINGHAM, Y. A., DARRITH, B. & VALLE, C. J. D. 2017. Serum albumin predicts survival and postoperative course following surgery for geriatric hip fracture. *Journal of Bone and Joint Surgery - American Volume,* 99**,** 2110-2118.

BOHL, D. D., SHEN, M. R., KAYUPOV, E., CVETANOVICH, G. L. & DELLA VALLE, C. J. 2016a. Is Hypoalbuminemia Associated With Septic Failure and Acute Infection After Revision Total Joint Arthroplasty? A Study of 4517 Patients From the National Surgical Quality Improvement Program. *Journal of Arthroplasty,* 31**,** 963-967.

BOHL, D. D., SHEN, M. R., KAYUPOV, E. & DELLA VALLE, C. J. 2016b. Hypoalbuminemia Independently Predicts Surgical Site Infection, Pneumonia, Length of Stay, and Readmission After Total Joint Arthroplasty. *Journal of Arthroplasty,* 31**,** 15-21.

CHEN, J., ZHENG, C. J., ZHONG, J. X., ZHAO, G. L., SHI, J. S., HUANG, G. Y., WEI, Y. B., WANG, S. Q., YU, J. & XIA, J. 2021. Preoperative prognostic nutritional index is useful factor for predicting postoperative delirium after primary total joint arthroplasty. *Bmc Musculoskeletal Disorders,* 22**,** 8.

COURTNEY, P. M., ROZELL, J. C., MELNIC, C. M., SHETH, N. P. & NELSON, C. L. 2016. Effect of Malnutrition and Morbid Obesity on Complication Rates Following Primary Total Joint Arthroplasty. *Journal of surgical orthopaedic advances,* 25**,** 99-104.

EMINOVIC, S., VINCZE, G., EGLSEER, D., RIEDL, R., SADOGHI, P., LEITHNER, A. & BERNHARDT, G. A. Malnutrition as predictor of poor outcome after total hip arthroplasty.

FU, M. C., D'AMBROSIA, C., MCLAWHORN, A. S., SCHAIRER, W. W., PADGETT, D. E. & CROSS, M. B. 2016. Malnutrition Increases With Obesity and Is a Stronger Independent Risk Factor for Postoperative Complications: A Propensity-Adjusted Analysis of Total Hip Arthroplasty Patients. *Journal of Arthroplasty,* 31**,** 2415-2421.

FU, M. C., MCLAWHORN, A. S., PADGETT, D. E. & CROSS, M. B. 2017. Hypoalbuminemia Is a Better Predictor than Obesity of Complications After Total Knee Arthroplasty: a Propensity Score-Adjusted Observational Analysis. *HSS Journal,* 13**,** 66-74.

HEIMROTH, J. C., NEUFELD, E. V., SODHI, N., WALDEN, T., WILLINGER, M. L. & BORAIAH, S. 2023. Relationship Between Preoperative Nutritional Status and Predicting Short-Term Complications Following Revision Total Hip Arthroplasty. *The Journal of Arthroplasty,* 38**,** 1326-1329.

HOLBERT, E. S., BRENNAN, J. C., JOHNSON, A. H., TURCOTTE, J. J., KING, P. J. & MACDONALD, J. H. 2023. The effects of hypoalbuminemia in obese patients undergoing total joint arthroplasty. *Archives of Orthopaedic and Trauma Surgery,* 143**,** 4813-4819.

HU, W., SONG, Z., SHANG, H., WANG, J. & HAO, Y. 2023. Inflammatory and nutritional markers predict the risk of post-operative delirium in elderly patients following total hip arthroplasty. *Frontiers in Nutrition,* 10.

HUR, E. S., BOHL, D. D., DELLA VALLE, C. J., VILLALOBOS, F. & GERLINGER, T. L. 2020. Hypoalbuminemia Predicts Adverse Events following Unicompartmental Knee Arthroplasty. *Journal of Knee Surgery,* 36**,** 491-497.

JOHNSON, N. R., STATZ, J. M., ODUM, S. M. & OTERO, J. E. 2021. Failure to Optimize Before Total Knee Arthroplasty: Which Modifiable Risk Factor is the Most Dangerous? *Journal of Arthroplasty,* 36**,** 2452-2457.

KAMATH, A. F., NELSON, C. L., ELKASSABANY, N., GUO, Z. G. & LIU, J. B. 2017. Low Albumin Is a Risk Factor for Complications after Revision Total Knee Arthroplasty. *Journal of Knee Surgery,* 30**,** 269-275.

KIEFFER, W. K. M., RENNIE, C. S. & HE, A. J. 2013. Preoperative albumin as a predictor of one-year mortality in patients with fractured neck of femur. *Annals of the Royal College of Surgeons of England,* 95**,** 26-28.

KURTOGLU, A., SUKUR, E., KOCHAI, A., SUKUR, N. E. O., OZ, I., KESKIN, D. & SEN, Z. 2021. Hypoalbuminemia Leads to An Increased Risk Of Mortality And Re-Operation Within 3-Months In Patients 75 < Years Of Age After Surgery For Hip Fractures. *Progress in Nutrition,* 23**,** 8.

LU, P., XIE, T., DAI, G., LI, Y., ZOU, J., CHEN, H. & RUI, Y. 2021. [Risk factors analysis for postoperative mortality of elderly patients with femoral neck fracture undergoing hemiarthroplasty]. *Chung-Kuo Hsiu Fu Chung Chien Wai Ko Tsa Chih/Chinese Journal of Reparative & Reconstructive Surgery,* 35**,** 217-220.

MAIMAITI, Z., XU, C., FU, J., LI, W. T., CHAI, W., ZHOU, Y. G. & CHEN, J. Y. 2021. A Novel Biomarker to Screen for Malnutrition: Albumin/Fibrinogen Ratio Predicts Septic Failure and Acute Infection in Patients Who Underwent Revision Total Joint Arthroplasty. *Journal of Arthroplasty,* 36**,** 3282-3288.

MAN, S. L., CHAU, W. W., CHUNG, K. Y. & HO, K. K. W. 2020. Hypoalbuminemia and obesity class II are reliable predictors of peri-prosthetic joint infection in patient undergoing elective total knee arthroplasty. *Knee Surgery & Related Research,* 32**,** 21.

MEYER, C. P., RIOS-DIAZ, A. J., DALELA, D., RAVI, P., SOOD, A., HANSKE, J., CHUN, F. K. H., KIBEL, A. S., LIPSITZ, S. R., SUN, M. & TRINH, Q. D. 2017. The association of hypoalbuminemia with early perioperative outcomes – A comprehensive assessment across 16 major procedures. *American Journal of Surgery,* 214**,** 871-883.

NELSON, C. L., ELKASSABANY, N. M., KAMATH, A. F. & LIU, J. B. 2015. Low Albumin Levels, More Than Morbid Obesity, Are Associated With Complications After TKA. *Clinical Orthopaedics and Related Research,* 473**,** 3163-3172.

NELSON, C. L., KAMATH, A. F., ELKASSABANY, N. M., GUO, Z. G. & LIU, J. B. 2019. The serum albumin threshold for increased perioperative complications after total hip arthroplasty is 3.0 g/dL. *Hip International,* 29**,** 166-171.

NEWMAN, J. M., SODHI, N., KHLOPAS, A., PIUZZI, N. S., YAKUBEK, G. A., SULTAN, A. A., KLIKA, A. K., HIGUERA, C. A. & MONT, M. A. 2020. Malnutrition increases the 30-day complication and re-operation rates in hip fracture patients treated with total hip arthroplasty. *HIP International,* 30**,** 635-640.

OAKLEY, C. T., KONOPKA, J. A., RAJAHRAMAN, V., BARZIDEH, O. S., MEFTAH, M. & SCHWARZKOPF, R. 2023. Does the geriatric nutritional risk index predict complication rates and implant survivorship in revision total joint arthroplasty? *Archives of Orthopaedic and Trauma Surgery,* 143**,** 7205-7212.

POPP, D., NIA, A., BIEDERMANN, G., SCHMOELZ, L., SILVAIEH, S., TIEFENBOECK, T. M., HAJDU, S. & WIDHALM, H. K. 2023. Predictive Validity of Mortality after Surgically Treated Proximal Femur Fractures Based on Four Nutrition Scores—A Retrospective Data Analysis. *Nutrients,* 15**,** 3357.

SCHWARTZ, A. M., WILSON, J. M., FARLEY, K. X., BRADBURY, T. L. & GUILD, G. N. 2020. Concomitant Malnutrition and Frailty Are Uncommon, but Significant Risk Factors for Mortality and Complication Following Primary Total Knee Arthroplasty. *Journal of Arthroplasty,* 35**,** 2878-2885.

SHANG, J., JIANG, S., GONG, J., ZHAO, G., SU, D. & WANG, L. 2023. Low albumin-to-fibrinogen ratio predicts adverse clinical outcomes after primary total joint arthroplasty: A retrospective observational investigation. *International Wound Journal,* 20**,** 3690-3698.

TAN, Y., JIANG, L. X., LIU, H. K., PAN, Z. Q., WANG, H. & CHEN, L. B. 2021. The effect of preoperative hypoalbuminemia on complications after primary hip arthroplasty. *Journal of Orthopaedic Surgery and Research,* 16**,** 9.

TANAKA, T. 2016. Factors predicting perioperative delirium and acute exacerbation of behavioral and psychological symptoms of dementia based on admission data in elderly patients with proximal femoral fracture: A retrospective study. *Geriatrics and Gerontology International,* 16**,** 821-828.

TIAN, Y. X., ZHU, Y. B., ZHANG, K. X., TIAN, M., QIN, S. H. & LI, X. T. 2022. Relationship Between Preoperative Hypoalbuminemia and Postoperative Pneumonia Following Geriatric Hip Fracture Surgery: A Propensity-Score Matched and Conditional Logistic Regression Analysis. *Clinical Interventions in Aging,* 17**,** 495-503.

TRAVERSO, G., NÚÑEZ, J. H., GEHRKE, T. & CITAK, M. 2023. Hypoalbuminemia increases the risk of failure following one-stage septic revision for periprosthetic joint infection. *Archives of Orthopaedic and Trauma Surgery,* 143**,** 5641-5648.

WALLS, J. D., ABRAHAM, D., NELSON, C. L., KAMATH, A. F., ELKASSABANY, N. M. & LIU, J. B. 2015. Hypoalbuminemia More Than Morbid Obesity is an Independent Predictor of Complications After Total Hip Arthroplasty. *Journal of Arthroplasty,* 30**,** 2290-2295.

WILSON, J. M., SCHWARTZ, A. M., FARLEY, K. X., BRADBURY, T. L. & GUILD, G. N. 2020. Combined Malnutrition and Frailty Significantly Increases Complications and Mortality in Patients Undergoing Elective Total Hip Arthroplasty. *Journal of Arthroplasty,* 35**,** 2488-2494.

YAO, W., TANG, W., WANG, W., LV, Q. & DING, W. 2023. Correlation between admission hypoalbuminemia and postoperative urinary tract infections in elderly hip fracture patients. *Journal of Orthopaedic Surgery and Research,* 18**,** 774.

YI, P. H., FRANK, R. M., VANN, E., SONN, K. A., MORIC, M. & DELLA VALLE, C. J. 2015. Is Potential Malnutrition Associated With Septic Failure and Acute Infection After Revision Total Joint Arthroplasty? *Clinical Orthopaedics and Related Research,* 473**,** 175-182.
