# Supplementary material for: Nutritional disorders have adverse effect on clinical outcomes and may increase healthcare costs following hip and knee arthroplasty—A systematic review
Source: J Exp Orthop. 2026 Oct 15;13(4):e70657. doi: 10.1002/jeo2.70657 (PMC13671359; doi:10.1002/jeo2.70657)
Supplement: Supplementary file 2 [file JEO2-13-e70657-s002.docx]

|  | **Design** | **Country** | **N=** | **Follow-up** | **Age (years)** | **Sex (% Female)** | **BMI (kg/m^2^)** | **Co-morbidity** | **Socio-economic status** |
| --- | --- | --- | --- | --- | --- | --- | --- | --- | --- |
| **Primary Total Hip** | | | | | | | | | |
| Cook et al. 2022a [8] | Retrospective cohort: CPRD  April 2009 - June 2019 | UK | 42,512 | 6 months | Median 71.4 (IQR 66.2-77.2) | 59.8 | / | / | Index Multiple Deprivation:  1: 12,359 (29.1%)  2: 11,009 (25.9%)  3: 9,078 (21.4%)  4: 6,278 (14.8%)  5: 3,788 (8.9%) |
| Cook et al. 2022b [9] | Retrospective cohort: CPRD  1998-1999 | UK | 103,563 | 90 days | 72.6 ± 7.5 | 61.5 | / | / | Index Multiple Deprivation incorporated into in regression model |
| Eminovic et al. 2021 [11] | Retrospective cohort over 8-year period (dates not specified) | Austria | 220 | 12 months | Overall: 76.3 ± 6.6  PEM vs non-PEM:  81.3 ± 7 vs 75.6 ± 6.2, p < 0.001 | PEM vs non-PEM: 71.1 vs 65.5  p=0.39 | Overall: 26.5 ± 4.3  PEM vs non-PEM: 26.8 ± 4.3 vs 24.7 ± 4.1 p=0.022 | Charlson Comorbidity Index: 1.8 ± 1.8  ASA: 1: 2 (0.9%) 2: 44 (20%) 3: 105 (47.7%) 4: 52 (23.6%)  Grade \| No-PEM \| PEM I \| 2 (100%) \| 0 II \| 44 (24.9%) \| 0 III \| 93 (52.5%) \| 12 (46.2%) IV \| 38 (21.5%) \| 14 (53.8%) p=0.001 | / |
| Fu et al. 2016 [12] | Retrospective cohort: NSQIP  2005-2013 | USA | 20,210 | 30 days | 18-64: 44.3%  65-79: 42.9%  80≤: 12.8% | 55.8 | 18.5-29.9: 54.4% 30-34.9: 25% 35-39.9: 12.7% 40+: 7.9% | Charlson Comorbidity Index:  0-2: 27.9%  3: 30.6%  4≤: 41.5% | / |
| Fu et al. 2017 [13] | Retrospective cohort: NSQIP  2005-2014 | USA | 34,800 | 30 days | 18-64: 39.2%  65-79: 49.6%  80≤: 11.2% | 63.2 | 18.5-29.9: 37.7% 30-34.9: 28.3% 35-39.9: 18.5% 40+: 15.5% | Charlson Comorbidity Index:  0-2: 20.9%  3: 32.3%  4≤: 46.8% | / |
| Hu et al. 2023 [17] | Retrospective cohort:  January 2019 - December 2022 | China | 254 | 7 days | 68.07 ± 5.68 | 65.7 | 24.84 ± 3.74 | / | / |
| Meessen et al. 2019 [35] | Retrospective analysis of prospective data  2012 - undetermined | Netherlands | 808  Frail 253  non-Frail 555 | 12 months | Frail vs non-Frail:  68.3 ± 10.3 vs 66.2 +/- 9.1  p=0.004. | Frail vs non-Frail:  74.2 vs 56.5  p<0.001 | Frail vs non-Frail: 28.1 ± 5.3 vs 26.6 ± 3.8  p<0.001 | Musculoskeletal Comorbidities (Severe back pain, Severe neck or shoulder pain, Severe elbow, wrist, or hand pain, Inflammatory arthritis, Other joint conditions)  **Frail vs non-Frail: 25% vs 12%, p<0.001**  Other Comorbidities (Asthma or chronic obstructive pulmonary disease, Cardiac disorder or coronary disease, Arteriosclerosis, Hypertension, Stroke, Severe bowel disorder, Diabetes mellitus, Migraine, Psoriasis, Chronic eczema, Cancer, Urine incontinence, Hearing or visual impairments, Dizziness in combination with falling). **Frail vs non-Frail: 84.1% vs 65%, p<0.001** | / |
| Meyer et al. 2017 [36] | Retrospective cohort: NSQIP  2005-2012 | USA | 15,074  Hypoalbuminaemia 764  Normal 14,310 | 30 days | As for combined | As for combined | As for combined | As for combined | / |
| Nanri el al. 2024 [37] | Retrospective cohort:  March 2017 - January 2022 | Japan | 356 | / | 73 (69-79) | 85.3 | 22.9 (20.8-25.1) | Charlson Comorbidity Index  0: 208 (58.4%)  1: 126 (35.4%)  2: 22 (6.2%)  3≤: 0 | / |
| Nelson et al. 2019 [39] | Retrospective cohort: NSQIP  2006 - 2013 | USA | 37,143  Hypoalbuminaemia 1570  Normal 35,573 | 30 days | Hypoalbuminaemia vs Normal:  68.4 ± 11.1 vs 67 ± 9.9  p<0.001 | Hypoalbuminaemia vs Normal:  69.9 vs 63.2  p<0.001 | / | Hypoalbuminemia vs Normal  ASA:  1: 0.57% vs 1.68% 2: 31.93% vs 50.57% 3: 67.5% vs 47.75%  p<0.001 | / |
| Okamoto et al. 2023 [42] | Retrospective case-control:  January 2018 - June 2021 | Japan | 205 | Mean: 3.6  Range: 2.0–5.5 | 72.2 ± 6.3 | 88.3 | 24.9 ± 3.4 | Charlson Comorbidity Index:  0: n=71  1-2: n=126  3≤: n=8 | / |
| Walls et al. 2015 [52] | Retrospective cohort: NSQIP  2006 - 2013 | USA | 24,238  Hypoalbuminaemia 1122  Normal 23,116 | 30 days | Hypoalbuminaemia vs Normal:  69.0 ± 13.7 vs 65.1 ± 11.7  p<0.01 | Hypoalbuminaemia vs Normal:  64.9 vs 55.2  p<0.01 | / | Hypoalbuminemia vs Normal  Charlson Comorbidity Index:  0: 69.9% vs 82.8% 1: 8.4% vs 4.4% 2: 15.2% vs 11.3% 3≤: 6.5% vs 1.5%  p<0.001  ASA:  1: 1.1% vs 3.6% 2: 30.6% vs 54.9%  3: 68.4% vs 41.5%  p<0.001 | / |
| Wilson et al. 2020 [53] | Retrospective cohort: NSQIP  2006 - 2018 (readmission, reoperation and discharge destination from 2011) | USA | 105,997  Hypoalbuminaemia 3291  Frail 14,781  Hypo + Frail 1078 | 30 days | Overall 64.9 ± 11.5  Healthy vs Hypoalbuminaemia vs Frail vs Hypo + Frail:  <54: 18.6% vs 17.3% vs 9.3% vs 7.2% 55-64: 31.1% vs 21.8% vs 27.7% vs 20.5% 65-74: 31.5% vs 28.8% vs 36.6% vs 31.7% 75≤: 18.9% vs 32.1% vs 26.4% vs 40.5%  p<0.001 | Overall: 54.5  Healthy: 45.2  Hypoalbuminaemia: 36.8  Frail: 49.5  Hypo + Frail: 41.2  p<0.001 | Overall: 30.4 ± 6.3  Healthy vs Hypoalbuminaemia vs Frail vs Hypo + Frail:  <18.5: 0% vs 0.8% vs 0.5% vs 1.8% 18.5-24: 19.9% vs 24.5% vs 10.1% vs 18.2% 25-29: 34.8% vs 29.9% vs 24.8% vs 22.6% 30-39: 38.1% vs 33.9% vs 50.6% vs 41.9% 40≤: 6.4% vs 9.4% vs 13.9% vs 15.5%  <0.001 | / | / |
| Zusmanovich et al. 2018 [61] | Retrospective cohort: NSQIP  2008 - 2015 | USA | 840  BMI <18.5 301  Normal 539 | 30 days | Underweight vs Normal:  71.6 vs 72.5  p=0.096 | Underweight vs Normal:  79.4 vs 78.1  p=0.726 | / | Underweight vs Normal  ASA 3≤: 128 (42.5%) vs 211 (39.1%) p=0.342. >10% weight loss: 1.7% vs 0% p=0.006 | / |
| **Primary Total Knee** | | | | | | | | | |
| Cook et al. 2022a [8] | Retrospective cohort: CPRD  April 2009 - June 2019 | UK | 49,208 | 6 months | Median 70.9 (IQR 66.3-75.8) | 55.5 | / | / | Index Multiple Deprivation:  1 (least deprived): 13,462 (27.4%)  2: 12,042 (24.5%)  3: 10,589 (21.5%)  4: 7,770 (15.8%)  5 (most deprived): 5,345 (10.9%) |
| Cook et al. 2022b [9] | Retrospective cohort: CPRD  1998-1999 | UK | 125,367 | 90 days | 72.3 ± 7.2 | 56.8 | / | / | Index multiple deprivation incorporated into in regression model |
| Humphrey et al. 2023 [18] | Retrospective case-control:  2016-2020 | USA | 140 | 1 year | / | 60.71 | / | / | / |
| Hwang et al. 2022 [20] | Retrospective cohort:  May 2018 - April 2021 | South Korea | 70 | / | Sarcopenia vs. non-Sarcopenia:  Unmatched: 74.5 ± 6.5 vs. 70.5 ± 6.6 p=0.001  Matched: 74.51 ± 6.46 vs. 74.19 ± 6.69 | Sarcopenia vs. non-Sarcopenia:  Unmatched: 94.3 vs. 86.1  Matched: 94.3 each | Sarcopenia vs. non-Sarcopenia:  Unmatched: 23.9 ± 3.4 vs 26.7 ±3.2 p<0.001.  Matched: 23.9 ±3.4 vs 24.1 ±2.60 | Sarcopenia vs. non-Sarcopenia  Charlson Comorbidity Index:  Unmatched:  0: 15 (52.9%) vs 191 (45.8%)  1: 12 (34.3%) vs 94 (22.5%)  2: 3 (8.6%) vs 58 (13.9%)  3≤: 5 (14.3%) vs 74 (17.7%)  p=0.462  Matched:  0: 15 (42.9%) vs 16 (45.7%)  1: 12 (34.3%) vs 8 (22.9%)  2: 3 (8.6%) vs 2 (5.7%)  3≤: 5 (14.3%) vs 9 (25.7%)  p=0.537 | / |
| Jin et al. 2024 [23] | Prospective observational: September 2020 - December 2021 | China | 164 | 3 months | 71.3 ± 4.6 | 76.2 | ≤23.9 = 20.7%  24-27.9 = 40.2%  28≤ = 39% | Charlson Comorbidity Index: 3 (2-4)  ASA 2: 7.3%  ASA 3: 92.7% | / |
| Johnson et al. 2021 [24] * | Retrospective cohort: NSQIP  2017-2018 | USA | 44,643 | 90 days | Mean 72  Range 68-77 | 61.7 | 40<: 10.03% | 4.4% active smokers  4.5% Insulin-dependent diabetes  14.6% Noninsulin dependent diabetes | / |
| Karamuri et al. 2023 [26] | Retrospective  cohort:  January 2020 - July 2021 | India | 435 | 1 year | Non-Vulnerable: 69.55 (range 65-83)  Vulnerable: 70.42 (range 65-86)  Frail: 76.10 (range 65-84)  p=0.067 | Non-Vulnerable: 63.48  Vulnerable: 52.88  Frail: 48.97 | Non-Vulnerable: 27.52 ± 4.25  Vulnerable: 28.57 ± 4.40  Frail: 31.40 ± 4.16  p=0.095 | Charlson Comorbidity Index:  Non-Vulnerable: 3 (2-4)  Vulnerable: 4 (2-5)  Frail: 5 (3-8) | / |
| Lakra et al. 2023 [30] | Retrospective  cohort: NRD  January 2017 - November 2019 | USA | 882,479 | 30-day | Frail vs non-Frail:  18-44: 0.5% vs. 1.0%  45-64: 24.6% vs. 37.6%  65-74: 39.2% vs. 39.9%  75≤: 35.7% vs. 21.5%  p<0.01 | Frail vs non-Frail:  66.2 vs. 62  p<0.01 | / | / | Frail vs non-Frail:  Medicare: 21.3% vs 20.7%  Medicaid: 3.7% vs 4.5%  Private: 19.75 vs 33.2%  Self-pay: 0.5% vs 0.4%  p<0.01 |
| Man et al. 2020 [34] | Retrospective cohort:  2013 - 2017 | Hong Kong | 624 | / | Hypoalbuminemia vs Normal:  68.9 ± 8.2 vs 67.3 ± 7.2 | Hypoalbuminemia vs Normal:  70.6 vs 75.5 | <18.5: 1.2% 18.5 - 22.9: 12.5% 23 - 24.9: 14.8% 25 - 29.9: 44.5% 30≤: 26.9% | Hypoalbuminemia vs Normal  Hypertension: 88.2% vs 69.9%  Renal Disease: 23.5% vs 3.1% Stroke/TIA: 5.9% vs 5.1% Rheumatological disease: 17.6% vs 4.4% Gouty arthritis: 17.6% vs 3.1%  Chronic liver disease: 0% vs 1.2% Hypothyroidism: 0% vs 3.8% Depression: 11.8% vs 2.5% Psychosis: 0% vs 0.3% History of DVT or PE: 0% vs 2.1% Chronic pulmonary disease: 0% vs 0.7% History of MI: 0% vs 5.3% Congestive Heart failure: 0% vs 0.5% Arrhythmia: 0% vs 3.1% Valvular Disease: 0% vs 0.8% Peptic ulcer disease 11.8% vs 0.5% Any malignancy: 0% vs 4.1% | / |
| Meessen et al. 2019 [35] | Retrospective analysis of prospective data 2012 - undetermined | Netherlands | 640  Frail 163  non-Frail 477 | 12 months | Frail vs non-Frail:  68.2 ± 8.7 vs 66.1 ± 8.6 p=0.01 | Frail vs non-Frail:  76.7 vs 61.5  p<0.001 | Frail vs non-Frail:  30.0 ± 5.2 vs 28.9 ± 4.4  p=0.022 | Musculoskeletal Comorbidities (Severe back pain, Severe neck or shoulder pain, Severe elbow, wrist, or hand pain, Inflammatory arthritis, Other joint conditions) **Frail vs non-Frail: 29.3% vs 21.6% p=0.054**  Other Comorbidities (Asthma or chronic obstructive pulmonary disease, Cardiac disorder or coronary disease, Arteriosclerosis, Hypertension, Stroke, Severe bowel disorder, Diabetes mellitus, Migraine, Psoriasis, Chronic eczema, Cancer, Urine incontinence, Hearing or visual impairments, Dizziness in combination with falling) **Frail vs non-Frail: 84.2% vs 70.5% p<0.001** | / |
| Meyer et al. 2017 [36] | Retrospective cohort: NSQIP 2005-2012 | USA | 23,856  Hypoalbuminaemia1118  Normal 22,738 | 30 days | As for combined | As for combined | As for combined | As for combined | / |
| Nanri el al. 2024 [37] | Retrospective cohort: March 2017 - January 2022 | Japan | 243 | / | 76 (71-80) | 75.7 | 25.2 (23.0-26.9) | Charlson Comorbidity Index:  0: 107 (44.0%)  1: 121 (49.8%)  2: 15 (6.2%)  ≥3: 0 | / |
| Nelson et al. 2015 [38] | Retrospective cohort: NSQIP 2006 - 2013 | USA | 37,143  Hypoalbuminaemia 1570  Normal 35,573 | 30 days | Hypoalbuminemia vs Normal:  68.4 ± 11.1 vs 67.0 ± 9.9  p<0.001 | Hypoalbuminemia vs Normal:  69.9 vs 63.2  p<0.001 | / | Hypoalbuminemia vs Normal  Charlson Comorbidity Index:  0: 68.41% vs 77.31% 1: 7.32% vs 3.67% 2: 20.19% vs 17.41% 3≤: 4.08% vs 1.62%   p<0.001 | / |
| Schwartz et al. 2020 [44] | Retrospective cohort: NSQIP 2006 - 2018 (2011-2018 for readmission and reoperation) | USA | 179,702 | 30 days | 66.4 ± 9.6 | 62 | 32.5 ± 6.6 | / | / |
| Shon et al. 2023 [47] | Retrospective cohort: May 2020 - August 2021 | Korea | 445 | Minimum 12 months | 71.8 (60–88) | 88.3 | 26.9 ± 3.5 | Modified Charlson Comorbidity Index:  Sarcopenia:  3 and 4: 3 (7.1%)  5–8: 20 (47.6%)  ≥9: 19 (45.2%)  Non-Sarcopenia:  2: 18 (4.5%)  3 and 4: 207 (51.4%)  5–8: 136 (33.7%)  ≥9: 42 (10.4%)  p<0.001 | / |
| **All-form Primary Hip (Total and Hemiarthroplasty)** | | | | | | | | | |
| Tan et al. 2021 [48] | Retrospective case-control:  January 2019 - December 2020 | China | 211  Hypoalbuminaemia; 93 (56 THA and 37 Hemiarthoplasty)  Control: 118 (75 THA and 43 Hemiarthroplasty) | / | Overall: 74.21 ± 8.46  Hypoalbuminemia vs Normal: 75.88 ± 8.92 vs 73.20 ± 8.04  p=0.026 | Hypoalbuminemia vs Normal:  63.75 vs 69.47 p=0.391 | Overall: 22.5 ± 2.4  Hypoalbuminemia vs Normal:  22.08 ± 2.03 vs 22.76 ± 2.57 p=0.045 | Hypoalbuminemia vs Normal  ASA  1: 0% vs 5.3%  2: 60% vs 67.9% 3: 40% vs 26.7%  p=0.023 | / |
| **Unicompartmental Knee** | | | | | | | | | |
| Hur et al. [19] | Retrospective cohort: NSQIP 2006-2018 | USA | 6049 | 30 days | / | / | / | Congestive Heart Failure: 9.1% Dyspnoea on exertion: 5.2% Hypertension: 3.1% End-stage renal disease: 11.1% COPD: 7.3% Current Smoker: 4.6% Anaemia: 5.8% | / |
| **Primary Total Hip and Knee (combined cohorts)** | | | | | | | | | |
| Babu et al. 2019 [1] | Retrospective case-control: 2015 - 2017 | USA | 99  THA 56  TKA 43  Low PLVI: 49  High PLVI: 50 | 1 year | Low vs High PLVI: 73.35 vs 68.08  p=0.015 | Low vs High PLVI: 59.18 vs 32  p=0.007 | Low vs High PLVI: 26.267 vs 31.247  p=0.013 | Low vs High PLVI  Charlson Comorbidity Index: 5.05 vs 3.84 p=0.009  ASA: 2.84 vs 2.46  p=0.003 | / |
| Blevins et al. 2018 [3] | Retrospective cohort:  2000-2016 | USA | 33,037  Hip 17,554  Knee 15,483 | 2 years | 63.48 ± 11.7 | 56.1 | 30.04 ± 6.8 | / | / |
| Bohl et al. 2016b [6] | Retrospective cohort: NSQIP 2011-2013 | USA | 49,603  Hip 19,975  Knee 29,628 | 30 days | / | / | / | / | / |
| Chen et al. 2021 [7] | Retrospective cohort:  January 2013 - October 2019 | China | 994  Hip 528  Knee 466 | 1 year minimum | Post-op Delirium vs Normal:  71.1 ± 9.6 vs 66.4 ± 9.7 | 71.2  (73.1 Post-op Delirium) | Post-op Delirium vs Normal:  24.2 ± 3.6 vs 23.8 ± 3.6  p=0.41 | Post-op Delirium vs Normal  ASA:  1-2: 59.7% vs 65.9% 3-4: 40.3% vs 34.1% | / |
| Courtney et al. 2016 [10] | Retrospective cohort:  June - December 2013 | USA | 670  Hip 236  Knee 434 | Medical = 90 days  Surgical = minimum 6 months | Hypoalbuminaemia: BMI >40: 57.0 BMI <40: 60.5  p=0.2  Normal: BMI >40: 59.4 BMI <40: 61.4  p=0.051 | / | Hypoalbuminaemia: BMI >40: 46.7 BMI <40: 29.4  Normal: BMI >40: 44.8 BMI <40: 29.6  p<0.001 for both | BMI >40 vs <40  Chronic Obstructive Pulmonary Disease: Normal: 3% vs 4% p=0.782 Hypoalbuminaemia 0% vs 10% p=0.175  Coronary Artery Disease: Normal: 7% vs 8% p=0.661 Hypoalbuminaemia: 16% vs 10% p=0.465  Congestive Heart Failure: Normal: 3% vs 2%, p = 0.443 Hypoalbuminaemia: 16% vs 10%, p = 0.465  Intraoperative Vasopressors: Normal: 30% vs 41%, p = 0.015 Hypoalbuminaemia; 42% vs 44%, p = 0.841  Chronic Kidney Disease: Normal: 7% vs 12%, p = 0.132 Hypoalbuminaemia: 17% vs 25%, p = 0.566 | / |
| Holbert et al. 2023 [16] | Retrospective cohort: NSQIP  2016-2020 | USA | 79,784  Hypoalbuminaemia 3959  Normal 75,825 | 30 days | Hypoalbuminaemia vs Normal:  64.79 ± 9.41 vs 63.35 ± 9.05  p<0.001 | Hypoalbuminaemia vs Normal:  72.5 vs 63.3  p<0.001 | Hypoalbuminaemia vs Normal:  35-40: 51.2% vs 61.4%  40-45: 30.1% vs 27.0%  45-50: 11.9% vs 8.3%  50≤: 6.8% vs 3.3%  p<0.001 for all | Hypoalbuminaemia vs Normal:  ASA 3≤: 79.7% vs 67.4%  p<0.001 | / |
| Meyer et al. 2017 [36] | Retrospective cohort: NSQIP 2005-2012 | USA | 38,930  Hip:  Hypoalbuminaemia 764  Normal 14,310  Knee:  Hypoalbuminaemia 1118  Normal 22,738 | 30 days | Overall: 67 (59-79)  Hypoalbuminaemia: 71 (61-79)  Normal: 67 (59-75)  p<0.001 | Hypoalbuminaemia: 49.12  Normal: 44.9 | Hypoalbuminaemia vs Normal:  <18.5: 5.39% vs 2.08%  18.5-24.9: 35.05% vs 24.46% 25-29.9: 30.68% vs 35.33%  30≤: 28.89% vs 38.12%  p<0.001 | Hypoalbuminemia vs Normal  ASA:  1: 0.57% vs 1.6% 2: 17.11% vs 34.88% 3: 65.84% vs 54.98%  4: 16.48% vs 8.54% | / |
| Shang et al. 2022 [46] | Retrospective cohort: 2016 - 2020 | China | 1532 | 66 months | 71.7 | 68.0 | Nutrition Risk Score  <3: 24.4 ± 3.3  3≤: 19.2 ± 3.6  p<0.001 | Charlson Comorbidity Index: 0 (1) vs 0 (1) | / |
| Shang et al. 2023 [45] | Retrospective cohort: January 2016 - June 2021 | China | 1791  Hips 1147  Knees 644 | 1, 3, and 6-months then yearly but no median or mean duration | 71.65 ± 10.59 | 68.3 | 24 ± 3.62 | Charlson Comorbidity Index: 0 (1) vs 0 (1) | / |
| **Revision Hip** | | | | | | | | | |
| Heimroth et al. 2023 [15] | Retrospective cohort: 2006-2019 | USA | 12,249 | 30 days | / | / | <18.5: 1.8%  18.5-29.9: 53.7%  30+: 44.5% | / | / |
| Tram et al. 2023 [51] | Retrospective cohort: NRD January 2017 - November 2019 | USA | 36,243  15,448 Dislocation  11,062 Mechanical  9733 Infection | 30 days | **Dislocation (Frail vs Non-Frail)**  18 - 44: 1.3% vs 3.2%  45 - 64: 19.3% vs 34.5%  65 - 74: 27.1% vs 32.7%  75≤: 52.3% vs 29.6%  p<0.01  **Mechanical (Frail vs Non-Frail)**  18 - 44: 1.2% vs 3.4%  45 - 64: 22.5% vs 37.9%  65 - 74: 30% vs 33.8%  75≤: 46.3% vs 24.9%  p<0.01  **Infection (Frail vs Non-Frail)**  18 - 44: 3% vs 6.1%  45 - 64: 32.7% vs 47.2%  65 - 74: 30.1% vs 29.8%  75≤: 34.3% vs 17%  p<0.01 | Frail vs non-Frail  Dislocation: 70.1 vs 63.9  Mechanical: 61 vs 52.9  Infection: 53.7 vs 45.9  p<0.01 for all | / | / | **Dislocation (Frail vs Non-Frail)**  Medicare: 83.9% vs 68.5%  Medicaid: 4.4% vs 6.1%  Private: 10% vs 22%  Self: 0.2% vs 0.6%  **Mechanical (Frail vs Non-Frail)**  Medicare: 79% vs 61.9%  Medicaid: 4.3% vs 5.3%  Private: 13.8% vs 29.2%  Self: 0.5% vs 0.6%  p<0.01  **Infection (Frail vs Non-Frail)**  Medicare: 70.3% vs 54.3%  Medicaid: 6.9% vs 8.9%  Private: 19.3% vs 32.4%  Self: 0.8% vs 1.2%  p<0.01 |
| **Revision Knee** | | | | | | | | | |
| Kamath et al. 2017 [25] | Retrospective cohort: NSQIP 2006-2014 | USA | 4551 | 30 days | Hypoalbuminaemia vs Normal  68.1 ± 11.8 vs 65.1 ± 10.8  p<0.001 | Hypoalbuminaemia vs Normal  56.6 vs 59.1 p=0.048 | / | Hypoalbuminaemia vs Normal  Charlson Comorbidity Index: 0: 62.365 vs 73.66% 1: 7.444% vs 5.137% 2: 24.58% vs 19.24% 3≤: 5.758% vs 2.034%  ASA:  1: 0.56% vs 0.83%, p < 0.001 2: 18.82% vs 40.47%, p < 0.001  3: 80.76% vs 57.57%, p < 0.001 | / |
| Kyaw et al. 2023 [29] | Retrospective cohort: NRD January 2017 - November 2019 | USA | 47,287  Instability: 9,458  Mechanical: 25,177  Infection: 12,712 | 30 days | Frail vs non-Frail  Instability  18-44: 0.7% vs 2%  45-64: 30.7% vs 42.7%  65-74: 36.5% vs 37%  75≤: 32.1% vs 18.3%  Mechanical  18-44: 0.7% vs 1.5%  45-64: 29.4% vs 42.6%  65-74: 36.4% vs 38.1%  75≤: 33.4% vs 17.9%  Infection  18-44: 1.2% vs 2.4%  45-64: 31.4% vs 41.9%  65-74: 37.4% vs 36.5%  75≤: 29.9% vs 19.2%  p<0.01 for all | Frail vs non-Frail  Instability  74.2 vs 64.1  Mechanical  65.5 vs 59.1  Infection  47.4 vs 44.1  p<0.01 for all | / | / | Frail vs non-Frail  Instability  Medicare 76.9% vs 61.4%  Medicaid 4.9% vs 5.4%  Private 14.8% vs 26.7%  Self 0.2% vs 0.3%  Mechanical  Medicare 73.7% vs 58.3%  Medicaid 3.3% vs 4.5%  Private 18.3% vs 31.1%  Self 0.3% vs 0.4%  Infection  Medicare 72% vs 59.5%  Medicaid 5.5% vs 7.1%  Private 18.4% vs 27.7%  Self 0.7% vs 0.7%  p<0.01 for all  p>0.05 for Income between frail and non-frail when stratified into quartiles |
| **Revision Hip and Knee (combined cohorts)** | | | | | | | | | |
| Bohl et al. 2016a [5] | Retrospective cohort: NSQIP  2005-2013 | USA | 4517  Septic 715  Aseptic 3802 | 30 days | Septic vs Aseptic  ≤24: 17.3% vs 18.9%  25-29: 32.3% vs 29.9%  30-34: 22.4% vs 25.4%  35-40: 13.7% vs 14.6%  40≤: 14.3% vs 11.2%  p=0.229 | Septic vs Aseptic  44.1 vs 59.2  p<0.001 | / | Septic vs Aseptic  Congestive Heart Failure: 98.6% vs 99.1%  p=0.238  Dyspnea on exertion: 91.8% vs 91.6% p=0.921  Hypertension: 68.8% vs 65.4%  p=0.076  Diabetes: 26.4% vs 15.9%  p<0.001  End-stage renal disease: 1.8% vs 0.34%  p<0.001  Chronic Obstructive Pulmonary Disease: 9.1% vs 6.4%  p=0.009  Smoker: 14% vs 12.9%  p=0.436 | / |
| Maimaiti et al. 2021 [33] | Retrospective cohort:  January 2017 - December 2019 | China | 466  Septic 259 (125 hips and 134 knees)  Aseptic 207 (153 hips and 54 knees) | 90 days | Septic vs Aseptic  60.83 ± 13.87 vs 61.28 ± 12.63  p=0.813 | Septic vs Aseptic  52.71 vs 59.42  p=0.148 | Septic vs Aseptic  25.62 ± 5.68 vs 25.11 ± 3.96  p=0.65 | Septic vs Aseptic  Charlson Comorbidity Index:  <4: 88.41% vs 86.67%  4≤: 11.59% vs 13.13%  p=0.618  ASA:  <3: 89.86% vs 88.42%  3≤: 10.14% vs 11.58% p=0.621 | / |
| Oakley et al. 2023 [41] | Retrospective cohort: 2011 -2021 | USA | 531 | Minimum 1 year | 73.1 [65.0–96.0] | 58.2 | 30.1 [16.1–53.1] | ASA:  1: n=3 (0.8%)  2: n=150 (37.5%)  3: n=223 (55.8%)  4: n=24 (6.0%) | / |
| Yi et al. 2015 [57] | Retrospective case-control:  2002 - 2010 | USA | 501  Hip 231  Knee 270  Septic 126  Aseptic 375 | 90 days | Septic vs Aseptic:  63.8 ± 12.5 vs 65.3 ± 11.8  p=0.2046 | Septic vs Aseptic: 53 vs 65  p=0.0258 | Septic vs Aseptic: 33.6 ± 8.7 vs 32.3 ± 8.2  p=0.2021 | Septic vs Aseptic  Charlson Comorbidity Index: 1.3 ± 1.7 vs 1 ± 1.5  p=0.1468 | Private vs non-private insurance with significantly increased rate of malnutrition in non-private patients:  OR 2.311 (1.182–4.516)  p = 0.0143 |
| **Hip Fracture** | | | | | | | | | |
| Bayram et al. 2021 [2] | Retrospective cohort:  2000-2019 | Turkey | 304  150 low PLVI  154 high PLVI | 35.7 ± 2.5 months alive  21.02 ± 2.4 months deceased | 79.3 ± 8.3 | 65.8 | / | ASA:  1: n=10  2: n=73  3: n=192  4: n=29 | / |
| Bohl et al. 2017 [4] | Retrospective cohort: NSQIP 2011-2013 | USA | 17,651 | 30 days | 65-69: 7.3%  70-74: 9.5%  75-79: 13.9%  80-84: 20.8%  85-90: 25.3%  90≤: 23.2% | 28.6 | <18: 8%  18-19.9: 10.6%  20 - 24.9: 41.1%  25 - 29.9: 27.2%  30 - 34.9: 9%  35≤: 4.2% | 809 HF  1619 Dyspnoea on exertion  12,748 Hypertension  408 end-stage renal disease  2354 COPD  1710 current smokers  11,960 anaemia | / |
| Guo et al. 2010 [14] | Prospective cohort:  September 2002 - December 2007 | China | 207 | 14-15 days | 75.93 ± 6.89 | 60.9 | Normal Wound Healing:  21.25 ± 2.70  Delayed Wound Healing: 19.53 ± 3.12  p < 0.01 | / | / |
| Kieffer et al. 2013 [27] | Retrospective analysis of prospective data collected 2008 | UK | 585  Hypoalbuminaemia 255  Normal 330 | 1 year | Median 84 | 77.9 | / | / | / |
| Kurtoglu et al. 2021 [28] | Retrospective cohort:  January 2017 - December 2019 | Turkey | 240 | 3 months | Hypoalbuminaemia vs Normal:  81.0 (68.0-97.0) vs 80.0 (75.0-96.0)  p=0.113 | Hypoalbuminaemia vs Normal:  67.3 vs 65.5 p=0.776 | Hypoalbuminaemia vs Normal:  24.5 (16.0-39.5) vs 26.6 (17.0-42.5)  p=0.003 | Hypoalbuminaemia vs Normal:  Charlson Comorbidity Index:  8.0 (4.0-13.0) vs 8.0 (4.0-12.0) p=0.006  ASA: 2: 8.5% vs 5.7% 3: 83% vs 93.1% 4: 8.5% vs 1.1% p=0.0423 | / |
| Lu et al. 2021 [32] | Retrospective cohort:  January 2011 - December 2015 | China | 109 | 2 years | / | 70.6 | / | / | / |
| Newman et al. 2020 [40] | Retrospective cohort: NSQIP January 2008 - December 2015 | USA | 1667  Hypoalbuminaemia 569  Normal 1098 | 30 days | Hypoalbuminaemia vs Normal:  73 ± 10.5 vs 71 ± 10.5  p=0.006 | Hypoalbuminaemia vs Normal:  64.5% vs 69.9% p=0.024 | Hypoalbuminaemia vs Normal:  23.9 vs 24.5 p = 0.134 | Hypoalbuminaemia vs Normal  Charlson Comorbidity Index: 0: 62.2% vs 74.6% 1: 28.1% vs 20.1% 2≤: 9.7% vs 5.3% p=0.0001  ASA:  <3: 16.5% vs 33.6% 3≤: 83.5% vs 66.4% p<0.0001  Smokers: 21.8% vs 14.5%, p < 0.0001  Independence Level: Independent: 81% vs 88% Partially Dependent: 15.6% vs 10.7% Totally Dependent: 2.6% vs 0.5% p<0.0001 | / |
| Popp et al. 2023 [43] | Retrospective cohort: January 2018 - November 2019 | Austria | 1080 | 12 months | Mean: 81.1 | 69.5 | Mean: 23.8 | / | / |
| Tanaka, 2016 [49] | Retrospective case-control:  November 2012 - March 2014 | Japan | 152  52 Delirium  100 Control | 1 week | Delirium vs Control:  86.5 ± 6.0 vs 83.1 ± 6.4  p=0.0015 | Delirium vs Control:  88.5% vs 80%  p=0.257 | Delirium vs Control:  20.4 ± 4.1 vs 20.6 ± 3.9  p=0.7577 | Delirium vs Control  ASA:  2 (1-3) vs 2 (2-3)  p=0.9025 | / |
| Tian et al. 2022 [50] - following propensity matching | Retrospective cohort:  January 2016 - December 2020 | China | 1318  Hypoalbuminaemia 659  659 Normal | / | Hypoalbuminaemia vs Normal:  76.8 ± 8.0 vs 76.9 ± 8.3 p=0.829 | Hypoalbuminaemia vs Normal:66.5 vs 68.4 p=0.445 | Hypoalbuminaemia vs Normal:  <18.5: 4.4% vs 5.5%  18.5-23.9: 64.8% vs 63.7% 24.0-27.9: 23.7% vs 24.0% 28.0≤: 7.1% vs 6.8%  p=0.833 | Hypoalbuminaemia vs Normal  ASA  I-II: 48.9% vs 51.4%  III-IV: 51.1% vs 48.6% p=0.349 | / |
| Winters et al. 2018 [54] | Prospective cohort:  November 2014 - November 2015 | Netherlands | 286 | Median: 25 months | 83 ± 6.6 | 75 | / | Cardiovascular disease 72%  Neurodegenerative disease 26%  Chronic pulmonary disease 11%  Diabetes Mellitus 22%  Cancer 9% | / |
| Yao et al. 2023 [56] | Retrospective cohort:  March 2013 - March 2023 | China | 1279 | / | Mean: 74.7 | 60.3 | / | ASA:  1-2: n=567 (44.33%)  3-4: n=712 (55.67%)  p<0.001 for correlation between decrease serum albumin and increased ASA | / |
| Zorrilla et al. 2004 [60] | Prospective cohort:  January 1998 - September 1999 | Spain | 97 | / | Mean: 80.2 (range 62–101) | / | / | / | / |
| **Primary and Revision Hip and Knee Arthroplasty (combined cohorts)** | | | | | | | | | |
| Ihle et al. 2017 [21] | Prospective cohort:  June 2014 - June 2015 | Germany | 192  Primary 170  Revision 22 | 13.7 ± 11.1 days normal 18.2 ± 11.7 days at-risk of malnutrition | 67.9 ± 10.7 | 58.3 | 28.1 ± 5.5 | 95.3% co-morbidities (62% cardiovascular disease but did study not specify which other conditions were counted) | / |
| Ihle et al. 2018 [22] | Prospective cohort:  June 2014 - June 2015 | Germany | 246  Primary 204  Revision 42 | 6 months | 67.9 (28-91) yrs  [mean of initial 351 patient but no difference from final 246 patients follow-up to 6 months] | F>M but numbers not given | / | / | / |
| Median (Range). Mean ± 1.96 SD. NSQIP = National Surgical Quality Improvement Program. ASA = American Association of Anaesthesiologists. CPRD = Clinical Practice Research database. * Data for all patients extracted from database not just those with albumin levels available. NRD = National Re-admission Database | | | | | | | | | |
