# Supplementary material for: Nutritional disorders have adverse effect on clinical outcomes and may increase healthcare costs following hip and knee arthroplasty—A systematic review
Source: J Exp Orthop. 2026 Oct 15;13(4):e70657. doi: 10.1002/jeo2.70657 (PMC13671359; doi:10.1002/jeo2.70657)
Supplement: Supplementary file 3 [file JEO2-13-e70657-s003.docx]

**AMED/EMBASE/Medline**

| 1 | Nutritional Status/ |
| --- | --- |
| 2 | ((status or questionnaire* or measure* or screen* or assess* or blood marker* or blood test* or serolog* or albumin or total lymphocyte* or transferrin or vitamin d or micronutrient*) adj3 (nutrition* or nutrient* or malnutrition* or malnourish* or undernourish* or immunonutri* or overnutrition* or overnourish* or sarcope* or sarcopae* or cachex* or cachec* or frail* or obese or obesity or metabol*)).mp. |
| 3 | 1 or 2 |
| 4 | (orthopaed* or orthoped* or arthroplast* or ((hip or joint or knee or spine or periarticular* or fractur*) adj3 (replace* or surger* or perioper* or postop* or post-op* or post op* or pre-op* or preop*))).mp. |
| 5 | exp orthopedics/ |
| 6 | 4 or 5 |
| 7 | 3 and 6 |
| 8 | limit 7 to yr="2000 -Current" |

**CENTRAL**

| ID | Search |
| --- | --- |
| #1 | MeSH descriptor: [Nutritional Status] explode all trees |
| #2 | MeSH descriptor: [Malnutrition] explode all trees |
| #3 | MeSH descriptor: [Obesity] explode all trees |
| #4 | MeSH descriptor: [Sarcopenia] explode all trees |
| #5 | MeSH descriptor: [Cachexia] explode all trees |
| #6 | MeSH descriptor: [Frailty] explode all trees |
| #7 | MeSH descriptor: [Metabolic Diseases] explode all trees |
| #8 | nutrition* |
| #9 | MeSH descriptor: [Overweight] explode all trees |
| #10 | #1 OR #2 OR #3 OR #4 OR #5 OR #6 OR #7 OR #8 OR #9 |
| #11 | MeSH descriptor: [Orthopedic Procedures] explode all trees |
| #12 | MeSH descriptor: [Arthroplasty] explode all trees |
| #13 | orthop* |
| #14 | #11 OR #12 OR #13 |
| #15 | #10 AND #14 with Publication Year from 2000 to 2022, with Cochrane Library publication date Between Jan 2000 and Sep 2022, in Trials |

**Scopus**

( ( ( ( "Nutritional Status" OR ( ( status OR questionnaire* OR measure* OR screen* OR assess* OR "blood marker*" OR "blood test*" OR serolog* OR albumin OR "total lymphocyte*" OR transferrin OR "vitamin d" OR micronutrient* ) W/3 ( nutrition* OR nutrient* OR malnutrition* OR malnourish* OR undernourish* OR immunonutri* OR overnutrition* OR overnourish* OR sarcope* OR sarcopae* OR cachex* OR cachec* OR frail* OR obese OR obesity OR metabol* ) ) ) ) AND ( orthopaed* OR orthoped* OR arthroplast* OR "Orthopedic Procedures" OR ( ( hip OR joint OR knee OR spine OR periarticular* OR fractur* ) W/3 ( replace* OR surger* OR perioper* OR postop* OR post-op* OR "post op*" OR pre-op* OR preop* ) ) ) ) ) AND ( LIMIT-TO ( PUBYEAR , 2022 ) OR LIMIT-TO ( PUBYEAR , 2021 ) OR LIMIT-TO ( PUBYEAR , 2020 ) OR LIMIT-TO ( PUBYEAR , 2019 ) OR LIMIT-TO ( PUBYEAR , 2018 ) OR LIMIT-TO ( PUBYEAR , 2017 ) OR LIMIT-TO ( PUBYEAR , 2016 ) OR LIMIT-TO ( PUBYEAR , 2015 ) OR LIMIT-TO ( PUBYEAR , 2014 ) OR LIMIT-TO ( PUBYEAR , 2013 ) OR LIMIT-TO ( PUBYEAR , 2012 ) OR LIMIT-TO ( PUBYEAR , 2011 ) OR LIMIT-TO ( PUBYEAR , 2010 ) OR LIMIT-TO ( PUBYEAR , 2009 ) OR LIMIT-TO ( PUBYEAR , 2008 ) OR LIMIT-TO ( PUBYEAR , 2007 ) OR LIMIT-TO ( PUBYEAR , 2006 ) OR LIMIT-TO ( PUBYEAR , 2005 ) OR LIMIT-TO ( PUBYEAR , 2004 ) OR LIMIT-TO ( PUBYEAR , 2003 ) OR LIMIT-TO ( PUBYEAR , 2002 ) OR LIMIT-TO ( PUBYEAR , 2001 ) OR LIMIT-TO ( PUBYEAR , 2000 ) )

**Web of Science**

| 7 | #3 AND #6 Limited to studies published 01-01-2000 to ..-09-2022 |
| --- | --- |
| 6 | #4 OR #5 |
| 5 | ALL="Orthopedic Procedures" |
| 4 | (KP=(orthopaed* OR orthoped* OR arthroplast* OR ((hip OR joint OR knee OR spine OR periarticular* OR fractur* ) NEAR/3 (replace* OR surger* OR perioper* OR postop* OR post-op* OR "post op*" OR pre-op* OR preop* ))) OR AB=(orthopaed* OR orthoped* OR arthroplast* OR ((hip OR joint OR knee OR spine OR periarticular* OR fractur* ) NEAR/3 (replace* OR surger* OR perioper* OR postop* OR post-op* OR "post op*" OR pre-op* OR preop* )))) |
| 3 | #1 OR #2 |
| 2 | (KP=((status OR questionnaire* OR measure* OR screen* OR assess* OR "blood marker*" OR "blood test*" OR serolog* OR albumin OR "total lymphocyte*" OR transferrin OR "vitamin d" OR micronutrient* ) NEAR/3 (nutrition* OR nutrient* OR malnutrition* OR malnourish* OR undernourish* OR immunonutri* OR overnutrition* OR overnourish* OR sarcope* OR sarcopae* OR cachex* OR cachec* OR frail* OR obese OR obesity OR metabol*)) OR AB=((status OR questionnaire* OR measure* OR screen* OR assess* OR "blood marker*" OR "blood test*" OR serolog* OR albumin OR "total lymphocyte*" OR transferrin OR "vitamin d" OR micronutrient* ) NEAR/3 (nutrition* OR nutrient* OR malnutrition* OR malnourish* OR undernourish* OR immunonutri* OR overnutrition* OR overnourish* OR sarcope* OR sarcopae* OR cachex* OR cachec* OR frail* OR obese OR obesity OR metabol*))) |
| 1 | ALL="Nutritional Status" |
